# Seed-Transmitted Bacteria and Fungi Dominate Juvenile Plant Microbiomes

**DOI:** 10.3389/fmicb.2021.737616

**Published:** 2021-10-22

**Authors:** David Johnston-Monje, Janneth P. Gutiérrez, Luis Augusto Becerra Lopez-Lavalle

**Affiliations:** ^1^MaxPlanck Tandem Group in Plant Microbial Ecology, Universidad del Valle, Cali, Colombia; ^2^International Center for Tropical Agriculture, Palmira, Colombia; ^3^Department of Plant Microbe Interactions, Max Planck Institute for Plant Breeding Research, Cologne, Germany

**Keywords:** plant microbiome, plant mycobiome, spermosphere, rhizosphere, endophyte, phyllosphere, core microbiome, seed microbiome

## Abstract

Plant microbiomes play an important role in agricultural productivity, but there is still much to learn about their provenance, diversity, and organization. In order to study the role of vertical transmission in establishing the bacterial and fungal populations of juvenile plants, we used high-throughput sequencing to survey the microbiomes of seeds, spermospheres, rhizospheres, roots, and shoots of the monocot crops maize (B73), rice (Nipponbare), switchgrass (Alamo), *Brachiaria decumbens*, wheat, sugarcane, barley, and sorghum; the dicot crops tomato (Heinz 1706), coffee (Geisha), common bean (G19833), cassava, soybean, pea, and sunflower; and the model plants *Arabidopsis thaliana* (Columbia-0) and *Brachypodium distachyon* (Bd21). Unsterilized seeds were planted in either sterile sand or farm soil inside hermetically sealed jars, and after as much as 60 days of growth, DNA was extracted to allow for amplicon sequence-based profiling of the bacterial and fungal populations that developed. Seeds of most plants were dominated by Proteobacteria and Ascomycetes, with all containing operational taxonomic units (OTUs) belonging to *Pantoea* and *Enterobacter*. All spermospheres also contained DNA belonging to *Pseudomonas*, *Bacillus*, and *Fusarium*. Despite having only seeds as a source of inoculum, all plants grown on sterile sand in sealed jars nevertheless developed rhizospheres, endospheres, and phyllospheres dominated by shared Proteobacteria and diverse fungi. Compared to sterile sand-grown seedlings, growth on soil added new microbial diversity to the plant, especially to rhizospheres; however, all 63 seed-transmitted bacterial OTUs were still present, and the most abundant bacteria (*Pantoea*, *Enterobacter*, *Pseudomonas*, *Klebsiella*, and *Massilia*) were the same dominant seed-transmitted microbes observed in sterile sand-grown plants. While most plant mycobiome diversity was observed to come from soil, judging by read abundance, the dominant fungi (*Fusarium* and *Alternaria*) were also vertically transmitted. Seed-transmitted fungi and bacteria appear to make up the majority of juvenile crop plant microbial populations by abundance, and based on occupancy, there seems to be a pan-angiosperm seed-transmitted core bacterial microbiome. Further study of these seed-transmitted microbes will be important to understand their role in plant growth and health, as well as their fate during the plant life cycle and may lead to innovations for agricultural inoculant development.

## Introduction

Over hundreds of millions of years, angiosperms have coevolved with microbes that helped them acquire nutrients, resist stress, and combat pathogens. Today, plants are considered to be holobionts, a community of microbes cooperating and coevolving with their host to stimulate its anatomy, physiology, development, immunity, behavior, and genetic variation (Rosenberg and Zilber-Rosenberg, 2016). Agricultural science began to appreciate the importance of these plant–microbe interactions with the discovery of soil-inhabiting arbuscular mycorrhizal fungi, which colonize about 90% of land plant species and aid in nutrient acquisition, and also by the realization that nodules on the roots of leguminous plants are powered by nitrogen-fixing bacteria (Johnston-Monje et al., 2021). Besides mycorrhizae and rhizobia, agriculture in some parts of the world has for many decades appreciated that other microbes may also play important roles in plant growth and productivity; for example in the 1970s, stem-inhabiting bacterial endophytes were discovered in Brazil (principally coordinated by EMBRAPA Agrobiologia scientist Johanna Döbereiner) to be important in the nitrogen economy of graminaceous grasses (Baldani and Baldani, 2005). It was not until the advent of high-throughput sequencing technologies at the beginning of this new millennium, however, that the immense diversity of plant-associated microbes began to be understood by the broader scientific community, highlighting the potential to discover many new beneficial plant-associated bacteria and fungi. As this exciting frontier of agricultural science continues to unfold, rational microbiome engineering to improve crop resilience and productivity will only become possible if the rules of microbiome function, provenance, transmission, assembly, and inheritance are elucidated (Vandenkoornhuyse et al., 2015; Busby et al., 2017; Arif et al., 2020).

Plant-inhabiting microbial populations may vary between host species or cultivar; organs or tissues or surfaces; developmental stage; geographic location; plant health; and even year sampled (Berg et al., 2016; Müller et al., 2016; Compant et al., 2019). Microbes in the soil immediately around roots (rhizosphere) are studied for their importance in breaking down organic materials and producing nutrients for plant absorption, while microbes inside the plant (endosphere) influence plant physiology and help control pathogens. Microbes in aboveground parts of the plants inhabit what is known as the phyllosphere, where again they mostly help their host by influencing physiology and controlling pathogens. Less studied, seeds and the area around the germinating seed (spermospheres) are increasingly being appreciated as microbial habitats contributing microbiota that can protect seeds against rotting in the soil, aid in germination, and increase seedling vigor (Nelson, 2018). Probably because of the legacy effects relating to the agricultural importance of soil-transmitted mycorrhizae and rhizobia, even today, agricultural science still largely believes the majority of plant-inhabiting microbes in phyllospheres, endospheres, and rhizospheres are acquired by horizontal transmission from soil (Bakker et al., 2013; Vandenkoornhuyse et al., 2015). For example, a foundational study describing the core root microbiome of *Arabidopsis* concluded that all of the plant’s rhizosphere and endosphere comes from the soil, although it should be noted that the seeds used were surface sterilized and no microbe-free substrate was included as a negative control (Lundberg et al., 2012). Besides soil, microbes are also believed to horizontally colonize plant surfaces and endospheres through contact with insects (Allard et al., 2018), dust, rain, and other plant surfaces (Bulgarelli et al., 2013). Unlike phyllospheres or rhizospheres, the inside of the host plant (endosphere) is a controlled habitat, requiring horizontally transmitted endophytes to find ways to enter through cracks, wounds, stomata, or complex signal-based mechanisms (Ibáñez et al., 2017).

In the last few decades, various publications began documenting the presence of non-pathogenic bacteria and fungi in and on seeds of many plant species (Truyens et al., 2015; Nelson, 2018). Evidence has also begun accumulating that vertical or seed transmission also significantly contributes to the plant microbiome (Li et al., 2019). As an example, our previous studies on the juvenile maize microbiome have found that bacterial seed endophytes can colonize other plant tissues, travel throughout the endosphere, and exit the roots to colonize the rhizosphere (Johnston-Monje and Raizada, 2011). We have also found that bacterial populations in maize seeds are a more important source of inoculum for juvenile root endobiomes, than is soil (Johnston-Monje et al., 2014), and that the most abundant bacteria in juvenile maize rhizospheres are vertically rather than horizontally transmitted (Johnston-Monje et al., 2016). A variety of other plant species, including rice, *Arabidopsis thaliana*, wheat, and tomato, have been shown to acquire at least some of their microbiome from their seeds (Nelson, 2018). If plants are truly holobionts that have survived and coevolved with microbes for hundreds of millions of years (Vandenkoornhuyse et al., 2015), it makes sense that their most important symbionts would be vertically transmitted through seed rather than gambling that all of the correct soil-dwelling microbes might be available at the germination site (Nelson, 2018). Vertical transmission may also give beneficial microbes the chance to establish founder populations and claim priority effects, helping define the microbiome of the plant from early on (Toju et al., 2018). Although much work needs to be done to better understand the importance of seed endophytes, it has been shown that they can aid in germination, provide protection from pathogens, and improve mineral nutrition and vigor of the seedling (Puente et al., 2009; Nelson, 2018; Li et al., 2019). It is troubling to think that because of the use of vegetative propagation in plants like cassava, potatoes, and strawberries, in addition to the phytosanitary standards requiring the physical and chemical disinfestation of botanical or vegetative seeds in order to have pathogen-free crops, the normal transmission of microbes from seeds to seedlings may have been interrupted by modern agriculture (Berg and Raaijmakers, 2018).

Regardless of provenance, with thousands of different species of microbe in the plant biome, how does one determine which are the most important to the plant’s well-being and productivity? In ecology, a positive relationship between a specie’s abundance and occupancy is considered a robust indication of its ecological importance (Gaston et al., 2000). These principles also function well in the study of plant microbial ecology, where microbial abundance and occupancy are important for the identification of core microbiomes (Shade and Stopnisek, 2019). The ecological importance of abundance is intuitively easy to understand. Sequence the bacterial populations in soybean root nodules, and by far the most abundant members observed are *Bradyrhizobium*, which are the preferred nitrogen-fixing endosymbionts of those plants (Sharaf et al., 2019). Watermelon cultivars which are susceptible to fusarium wilt, will accumulate much higher levels of *Fusarium* in their roots than will resistant cultivars (Xu et al., 2020). Identify soils with high levels of plant pathogens (such as *Fusarium solani*, *Verticillium dahliae*, *Rhizoctonia solani*, and *Colletotrichum truncatum*), and it is possible to identify the part of the field the sickest strawberry plants will develop (Mirmajlessi et al., 2018).

When trying to identify a core microbiome, occupancy (how often a microbe is observed in a sample) is most often considered the defining characteristic. For example, one definition of a core microbiome are those bacteria and fungi that are closely associated with a particular species or genotype of plant (i.e., high occupancy), independent of environmental conditions (Toju et al., 2018). Core microbiomes are thought to contain key microbial taxa that have been important for plant survival and reproduction over evolutionary time (Shade and Handelsman, 2012; Lemanceau et al., 2017). Such microbes, must have over millions of years, developed a robust and efficient transmission strategy and retained the ability to colonize the plants and also to provide beneficial functions that contribute to plant growth, survival, and/or reproduction; traits which could be under positive selection in the holobiont (Wassermann et al., 2019a). Because of the theoretical importance for agriculture, searches for core microbiomes have been attempted in *Arabidopsis* (Lundberg et al., 2012; Brachi et al., 2017), potato (Pfeiffer et al., 2017), grape (Zarraonaindia et al., 2015), sugarcane (Hamonts et al., 2018), tomato (Lee et al., 2019), wheat (Schlatter et al., 2020), switchgrass (Bowsher et al., 2020), and rice (Edwards et al., 2015) among others. Beyond being important in the microbiome of a single plant species, microbes that are core to multiple plant species may be evidence of a larger pattern of transmission, environmental inoculation, or host evolution. It is interesting to speculate that the holobiont common ancestor of angiosperm plants, which split into monocots and dicots about 150 MYA (Chaw et al., 2004), would have also possessed a core microbiome that may have been passed on to all of its descendants. A few searches for core microbiomes across plant species have already been conducted, for example among the germinating seeds of 28 different species of agricultural plant including *A. thaliana*, *Solanum lycopersicum*, and *Phaseolus vulgaris* (Barret et al., 2015), among soil-grown roots of three *A. thaliana* ecotypes and wild relatives (Schlaeppi et al., 2014), among the roots and rhizospheres of 30 different crop plants grown from surface-sterilized seed in soil (Fitzpatrick et al., 2018), and inside the roots of 31 taxonomically diverse plant species growing on sand dunes in an Australian nature reserve (Yeoh et al., 2017). From a technological point of view, microbes with high occupancy and beneficial bioactivity across a spectrum of plant species are very attractive, theoretically allowing one strain to become an inoculant for diverse crop species. For example, *Burkholderia phytofirmans* is an endophytic bacteria that can colonize and promote the growth of a wide range of angiosperms including *A. thaliana*, grape, maize, potato, switchgrass, tomato, and wheat (Afzal et al., 2019). Another bacterial endophyte, *Gluconacetobacter diazotrophicus*, is claimed by the company Azotic Technologies to be able to both colonize and fix substantial amounts of nitrogen in a wide variety of plants including rice, wheat, maize, tomato, potato, tobacco, cotton, sunflower, lettuce, cassava, soybean, pea, beans, and even *A. thaliana* (Dent et al., 2017). The fungal endophyte *Piriformospora indica* has been tested on over 150 different species of plants, where it has consistently been shown to promote plant growth and enhance yield, increase seed germination and vigor, increase flowering/fruiting, augment nutrient uptake, and aid in abiotic and biotic stress resistance (Singhal et al., 2017).

Beneficial microbes are usually discovered in the lab and then screened in small-scale assays within labs or greenhouses. After isolation and screening, the strain then needs to be properly formulated for delivery into a farmer’s field if it ever hopes to impact agriculture, and this bottleneck can dramatically reduce the number of candidates that find commercial success. The most efficient and practical method of agricultural microbe delivery is through the seed, where a relatively small amount of inoculum is needed (compared to soil) and microbes are well positioned to colonize the emerging seedling and potentially the whole plant for its entire life (O’Callaghan, 2016). *B. phytofirmans* for example, was originally isolated from onion roots (Sessitsch et al., 2005), and despite consistently being able to promote growth of a wide variety of plants under controlled conditions, without development of an effective, practical, and scalable way to coat it onto crop seeds, this bacteria has not been able to directly impact agriculture as a commercial product. Rather than attempting to develop inoculant formulations to help root, shoot, rhizosphere, or soil microbes survive on the seed surface, the study of seed microbiomes from a variety of crop species may yield insights into which microbes are already best suited/preadapted to be seed inoculants (von-Maltzahn et al., 2015, 2017). The ideal situation for a company would be to find core microbes, adapted to life on the dry surface of a seed and with the ability to survive there for weeks or months until germination in farm soil, whereupon it can colonize the developing roots and shoots of any crop plant and begin to influence plant growth and health in a beneficial manner.

Our experiment attempts to document the common (appearing in over 60% of samples) and core (appearing in 100% of samples) microbes inhabiting seed interiors and seed surfaces (spermospheres) of a panel of 17 academically and economically important plant species, many of which have had their genomes sequenced and serve as model organisms. These plants include the monocot crops maize (*Zea mays* ssp. mays var. B73), rice (*Oryza sativa* ssp. japonica var. Nipponbare), switchgrass or Panicum (*Panicum virgatum* var. Alamo), *Brachiaria decumbens*, wheat (*Triticum aestivum*), sugarcane (*Saccharum officinarum*), barley (*Hordeum vulgare* ssp. vulgare), and sorghum (*Sorghum bicolor* ssp. bicolor); the dicot crops tomato (*S. lycopersicum* Heinz 1706), coffee (*Coffea arabica* var. Geisha), common bean (*P. vulgaris* G19833), cassava (*Manihot esculenta*), soybean (*Glycine max*), pea (*Pisum sativum*), and sunflower (*Helianthus annuus*); and the model plants *A. thaliana* (Columbia-0) and *Brachypodium distachyon* (Bd21). In order to try to see how much of the seed microbiome goes on to make up the microbial populations of developing plants, these were planted in sealed jars filled with sterile sand and water, then left to develop up to 2 months until harvesting their rhizospheres, root endospheres, and phyllospheres for DNA extraction. As soil is classically considered to be the most important source of a plant’s microbiome, the sealed jar experiment was also carried out using soil from a cassava field at the International Tropical Agriculture Research Institute in Colombia. Microbiomes of all sample types of plant species growing on sterile or non-sterile soil were compared bioinformatically based on sequencing of the bacterial 16S and fungal internal transcribed spacer (ITS). The primary purpose of this study was to demonstrate the importance of seed transmission to the establishment of plant microbiomes, which have traditionally been assumed to acquire all their microbes from soil. Showing that seeds are dominant players in establishing plant microbiomes could lead to a paradigm shift in our understanding (and ability to manipulate) of plant microbiome assembly, which has been assumed to depend largely on soil. A secondary purpose of this study was to establish whether core seed-transmitted microbiomes might exist across these economically important plant species. Core seed-transmitted microbes are evidence of evolutionary conservation and may point to important physiological functions these microbes perform for angiosperm seeds, as well as suggesting the existence of microbes that could function as broad host range inoculants in agriculture.

## Materials and Methods

### Sources of Seed

Seventeen different seed accessions were obtained for this experiment. From the U.S. National Plant Germplasm System of the U.S. Department of Agriculture were obtained (with accession numbers in brackets) the following: *H. annuus* var. Arrowhead (PI 650649), *H. vulgare* ssp. vulgare var. Beaver (CIho 1915), *O. sativa* ssp. japonica var. Nipponbare (GSOR 100), *P. virgatum* var. Alamo (PI 422006 01 SD), *P. sativum* var. Aa134 (PI 269818), *S. bicolor* ssp. bicolor var. BTx623 (PI 564163 02 SD), *T. aestivum* var. Prospect (PI 491568 TR04ID), and *Z. mays* ssp. mays var. B73 (PI 550473).

*Brachiaria decumbens* var. Basilisk (CIAT606), *M. esculenta* var. 19 (DI-2015), and *P. vulgaris* var. G19833 were obtained from the CIAT Genebank (Palmira, Valle del Cauca, Colombia).

*Solanum lycopersicum* var. Heinz 1706 (LA4345) was graciously provided by the C.M. Rick Tomato Genetics Resource Center (Davis, CA, United States).

*Arabidopsis thaliana* var. Columbia-0 and *B. distachyon* var. Bd21 were donated by the Hazen lab at the University of Massachusetts (Amherst, MA, United States).

*Saccharum officinarum* var. CS#725 (CC93-4112 x CC91-1987) was obtained from Cenicaña (Florida, Valle del Cauca, Colombia).

*Glycine max* var. Paramo 29 was purchased from Semillas del Pacifico (Cartago, Valle del Cauca, Colombia).

*Coffea arabica* var. Geisha was purchased from Agro Ingenio (El Chantaduro, Valle del Cauca, Colombia).

### Sources of Soil

#### Sterile Sand

River sand was purchased in bulk from a hardware store in Palmira, Colombia, and manually sieved to a uniform consistency using a 500-μm metal sieve. Sand was then sterilized by autoclaving twice for 20 min at 121°C, and after transfer to glass jars, it was autoclaved a third time for 20 min at 121°C.

#### Field Soil

**FIGURE 1:**
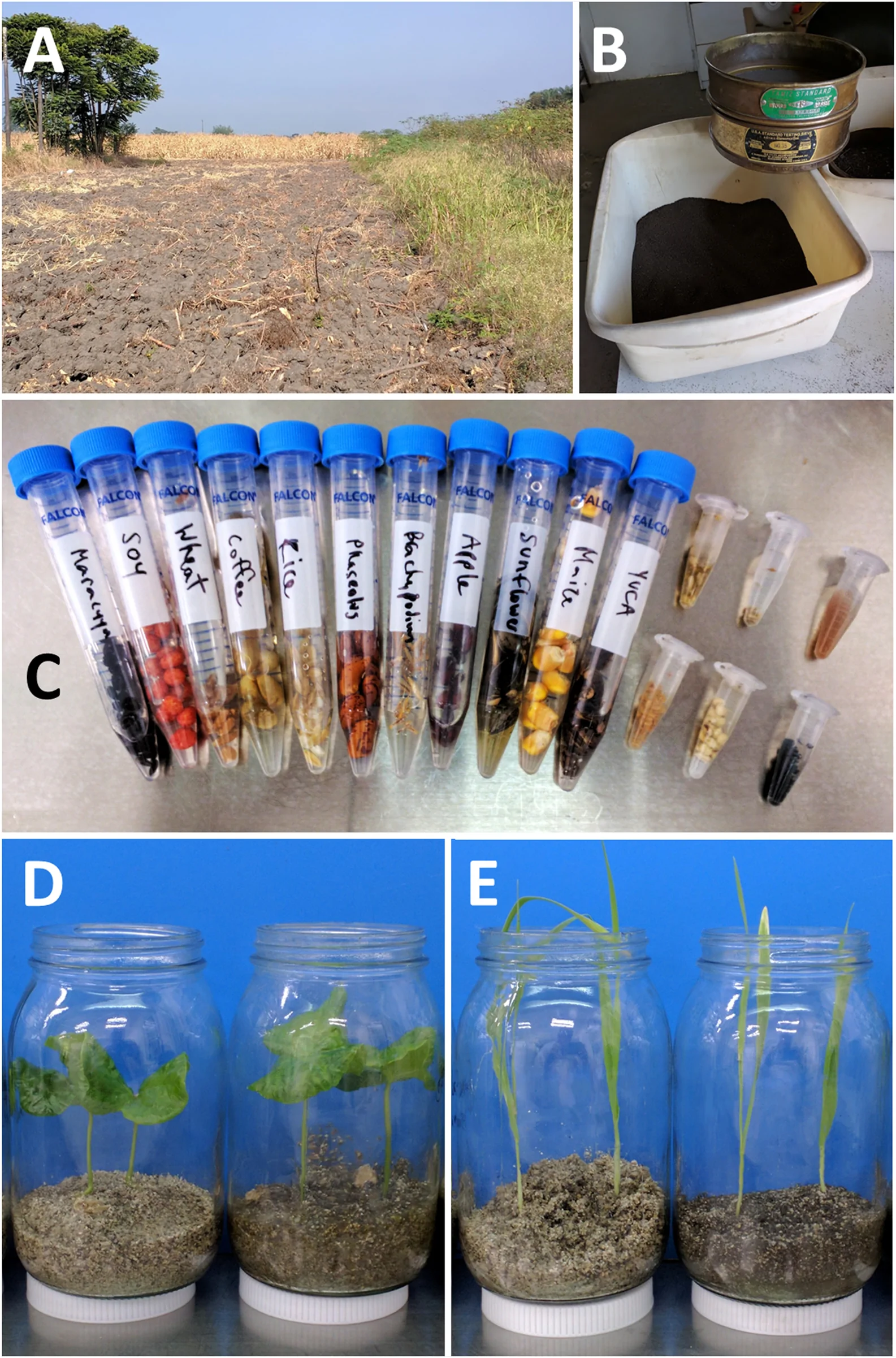
Soil, seeds, and examples of the gnotobiotic terrariums used to grow plants in this study. **(A)** Fallow cassava field at CIAT where soil was harvested. **(B)** Cassava field soil after sieving. **(C)** Soaking in sterile water prior to extraction of seed endosphere and spermosphere DNA (yucca = cassava; maracuya and apple were later replaced with pea and barley). **(D)** Coffee at harvest after growth in sterile sand on the left and field soil mixed with sand on the right. **(E)** Barley at harvest after growth in sterile sand on the left and field soil mixed with sand on the right.

An agricultural mollisol was excavated from a fallow cassava field at a CIAT property near Palmira, Colombia, at GPS coordinates 3.498434, –76.354959 (Figure 1A). Large clods were broken into smaller fragments by crushing and then manually sieving to a uniform consistency using a 500-μm metal sieve (Figure 1B).

Sieved sand and soil were both submitted for physio-chemical analysis by the “Suelos y Paisajes para la Sostenibilidad” group at CIAT Headquarters (Supplementary Table 1: Physiochemical Soil Properties).

### Experimental Setup and Plant Growth Conditions

Twenty large seeds or 0.5 g of small seeds of each accession were put in sterile 15- or 2-ml tubes and soaked for 6 h in double-distilled sterile water (Figure 1C). Half of these were then transferred to a sterile Petri dish containing a sterile Whatman #1 filter paper (GE HealthCare: United States) and irrigated with 3 ml of sterile water, while the other half received 3 ml of sterile water mixed with 1 g of field soil. These were incubated at 32°C in the dark for several days until germination.

From each dish, two seedlings were transplanted to corresponding jars as they germinated. Autoclaved glass jars were 13-cm tall, 7-cm wide in diameter, and filled with 100 ml of sterile sand (then autoclaved again) or with 100 ml of 1:1 soil/sterile sand, then watered once with 10 ml of sterile distilled water and sealed with a plastic lid. Jars were incubated (and never opened) in a single Panasonic MLR-352H Plant Growth Chamber set at 28°C for 12 h with 5 lm of fluorescent light and for 12 h of darkness at 22°C. Plants were grown between 2 weeks and 2 months, until they were of a significant size or until they hit the lid of the jar. Before harvesting, jar lids were removed inside a laminar flow hood, and shoots were allowed to dry off for 24 h (Figures 1D,E).

### Harvesting Seed and Root Endospheres, Spermospheres, Phyllospheres, and Rhizospheres

To collect spermospheres and seed endospheres, 2 (maize, Phaseolus, and sunflower), 5, or 0.1 g (Arabidopsis, Brachiaria, and sugarcane) seeds of each species were placed in a 15-ml conical tube and soaked in 5 ml of double-distilled autoclaved water, in darkness for 48 h at 32°C. Tubes were then shaken vigorously by hand and vortexed to dislodge microbes from seed surfaces, and then, supernatant liquid was decanted off into sterile conical tubes as spermosphere samples that were immediately frozen at –80°C. The remaining seeds were then surface sterilized/cleaned of DNA by soaking with agitation for 30 min in full-strength Chlorox bleach (6% Na_2_HPO_4_); rinsed three times in sterile, double-distilled water; and frozen at –80°C.

To collect shoot/phyllosphere material using sterile forceps and scissors, each plant was clipped just above where it emerged from the sand or soil, any remaining seed coat removed, transferred whole to a sterile 50-ml conical tube, cut into smaller pieces within the tube using sterile scissors, and then frozen at –80°C. To collect rhizosphere material, unwashed roots that had been excavated and separated with scissors from the shoot were shaken free of any attached soil and placed into sterile 50-ml conical tubes. To these, 10 ml of sterile distilled water was added and shaken, with the resulting “muddy wash” collected in a separate 15-ml conical tube as the rhizosphere, which was immediately frozen at –80°C. The roots continued to be rinsed several more times with sterile distilled water until both the wash and root surfaces were completely clean and clear, cut into smaller pieces within the tube using sterile scissors, and then frozen at –80°C in fresh 50-ml conical tubes for later processing (Johnston-Monje et al., 2017). No surface sterilization with aggressive chemicals was attempted on either root or shoot material, just vigorous washing with distilled water.

For each of the 17 species, three repetitions/jars per substrate were sampled (pooling the two plants inside each jar) for root, shoot, and rhizosphere (306 samples). Two repetitions of seed endospheres and spermospheres from each species were also harvested (68 samples).

### Sample Preparation and DNA Extraction

After thawing, rhizosphere and spermosphere washes were concentrated by centrifugation at 15,000 *g* for 15 min, generating a pellet. The supernatant was removed, and the process repeated until 3 ml of sample had been processed. The pellet was re-suspended in an additional 1 ml of spermosphere or rhizosphere wash, before proceeding with DNA extraction. In contrast, after thawing, 50-ml conical tubes containing roots, shoots, or seeds received five 6.35-mm carbon steel ball bearings and 1 ml of sterile distilled water and were then vigorously shaken by hand until the supernatant obtained the consistency of a thick soup.

Then, 400 μl of these liquid samples was transferred to a 2-ml Eppendorf tube containing five 2.3-mm zirconia/silica beads (Cat#11079125z, Biospec Products, United States) along with 500 μl of Qiagen Powerbead solution, RNAse A, Phenolics Blocker, and Solution SL (Qiagen, United States). These were shaken for 20 min in a Harbil 5G-HD 5 Gallon Shaker (Part#32940, Fluid Management, United States) and then centrifuged at 13,000 RCF for 2 min before up to 700 μl was aspirated off with a pipette and added to buffer IL. The rest of the protocol was followed as per Qiagen instructions with the DNeasy PowerPlant Pro HTP 96 Kit (Qiagen, United States).

### Metagenomic Sequencing Library Preparation

In order to prepare 16S and ITS amplicons for sequencing on the Illumina MiSeq platform, a two-step PCR strategy was employed, first amplifying all 380 DNA extracts with bacterial 16S primers and fungal ITS primers (768 PCR reactions) before dual labeling them with index sequences. The initial PCR was performed with an equimolar mix of staggered universal bacterial 16S [515FB and 806RB (Lundberg et al., 2013)] or fungal ITS [ITS1F and ITS2R (Smith et al., 2020)] primers that included 19 or 20-bp 5′ tail sequences complementary to Illumina MiSeq indexing primers (Supplementary Table 2). Anti-chloroplast (5′-GGCTCAACCCTGGACAG-3′) and anti-mitochondria (5′-GGCAAGTGTTCTTCGGA-3′) peptide nucleic acid (PNA) blockers were added to the bacterial 16S PCR reactions to block amplification of chloroplast and mitochondria as previously described (Lundberg et al., 2013). In a total volume of 25 μl, reactions were setup with 18.3 μl of nuclease-free water, 4 μl of 5X Phusion HF buffer, 0.4 μl of 10 mM dNTPs, 0.4 μl of each forward and reverse primer at 10 mM, 0.2 μl of BSA, 0.1 μl of Phusion enzyme (NEB, United States), 0.4 μl of each PNA blocker, and 0.5 μl of template DNA (concentration unknown). Reaction conditions were 35X (denaturation at 98°C for 10 s, PNA annealing at 81°C for 10 s, primer annealing at 50°C for 10 s, and elongation at 72°C for 20 s), final elongation at 72°C for 5 min, and then a cooldown to 4°C.

Without checking for amplification success, PCR product from each of the first 768 PCR reactions was used in a second PCR whose purpose was to dual-label amplicons and add flow cell adapter sequences. These 768 different labeling reactions were conducted using 24 different forward primers (TruSeq_F), containing unique 6-bp index sequences, and 32 different reverse primers (TruSeq_R), each containing unique 6-bp index sequences (Supplementary Table 2). In a total volume of 25 μl, step 2 reactions were setup with 19.2 μl of nuclease-free water, 4 μl of 5X Phusion HF buffer, 0.4 μl of 10 mM dNTPs, 0.4 μl of each TSf and TSr primers at 10 mM, 0.1 μl of Phusion enzyme (NEB, United States), and 0.5 μl of unpurified PCR product from step 1 (concentration unknown). Reaction conditions were initial denaturization at 98°C for 30 s, 15X (denaturization at 98°C for 10 s and primer annealing + elongation at 72°C for 20 s), final elongation at 72°C for 5 min, and then a cooldown to 4°C.

The products of these 768 labeling reactions were checked visually for successful amplification (bacterial 16S of 428 bp and fungal ITS of 470–525 bp) on 1% agarose gels and quantity estimated using ImageJ (Schneider et al., 2012) (note: except for negative controls of water and sterile sand, which did not amplify, unsuccessful PCR reactions were repeated until there was sufficient amplicon to allow approximately equimolar amounts of all 96 labeling reactions/plate to be pooled). With this software-assisted visual estimate of amplicon quantity for each reaction within a 96-well plate, equimolar amounts of each PCR product was mixed into eight pools. Pools were concentrated with ethanol precipitation and resuspension in 10% their volume of pure water. To purify target amplicons, 200 μl of each of the eight concentrated pooled sets of PCR products were run on a 2% agarose gel, the appropriate bands excised with a scalpel, and then gel fragments extracted with an Omega Bio-Tek E.Z.N.A. gel extraction kit (Norcross, Georgia, United States). The eight purified pools were again checked visually for purity on an agarose gel, quantified using the Picogreen dsDNA quantitation assay (ThermoFisher Scientific, United States), and sent for super-pooling and sequencing on a single 2 × 300-bp paired-end run on the Illumina MiSeq platform at a commercial sequencing facility (GENEWIZ, NJ, United States).

### Bioinformatics

MiSeq data was demultiplexed by the commercial sequencing facility and received as one FastQ file per sample, which have been deposited at the NCBI Sequence Read Archive (SRA) under BioProject PRJNA731997. Further sequence processing was done using USEARCH 11 using the recommended settings^1^. Briefly, paired-end reads were aligned and merged to form full-length sequences called “Uniques,” while quality filtering was performed to remove unmatched and low-quality reads. Next, the program binned these full-length reads together at a similarity threshold of 97% and formed a reference sequence for each bin referred to as an operational taxonomic unit (OTU). Only OTUs represented by two or more raw reads were used for analysis. Bacterial 16S OTUs were assigned a taxonomic identity by USEARCH trained on the RDP training set v16 (13,000 sequences), while fungal ITS OTUs were annotated by RDP Classifier (Cole et al., 2014) trained on the RDP Warcup training set v2 (18,000 sequences). Rarefaction of OTU counts was also performed with USEARCH 11. OTU annotations, total counts, and rarefied counts were exported to Excel (Microsoft, United States) for further analysis and visualization, with statistics done by XLSTAT (Addinsoft, France) and PAST 4^2^. Based on taxonomic annotation, OTUs with lower than 15% identity to a target sequence were hand checked and excluded if they were chloroplasts, mitochondria, plant ribosomes, protists, or other non-target sequences. OTU counts were normalized by transformation into proportional abundance as recommended elsewhere (McKnight et al., 2019).

## Results

### Plant Growth

After collecting and sieving sand and soil (Figures 1A,B), then soaking seeds from many different sources (Figure 1C), germinating seeds were planted in sealed jars with either sterile sand or field soil and left to grow for up to 2 months until they were harvested for DNA extraction. Figure 1D shows coffee plants growing in sterile sand and field soil, while Figure 1E shows barley.

### Sequencing Summary

A total of 8,294,046 merged bacterial 16S sequence pairs were obtained; however, after quality control, 5,370,471 high-quality reads remained that were binned at 97% sequence identity into 1,178 OTUs. Manual inspection of OTU taxonomy revealed 102 non-target OTUs that were chloroplast or plant/fungi mitochondria, so these were discarded, resulting in 4,945,887 remaining reads. A total of 377 bacterial samples with 16S amplicons yielded read count data, ranging from 12 for maize soil shoot #2 to 80,970 for *Brachypodium* spermosphere #2. Bacterial 16S read counts averaged about 13,015 per sample, compared to the average of 3,119 non-target reads per sample, which were bioinformatically removed. To compare the diversity in different samples using Bray-Curtis dissimilarity, we rarefied the data to 3,500 reads per shoot, 3,000 reads per root, and 6,500 reads per rhizosphere (although samples with low OTU counts were included without rarefaction); OTU counts were transformed to relative proportions, then averaged across repetitions. For all other figures and tables, reads from different reps were summed together, then transformed to relative proportion.

For fungal ITS, a total of 10,269,421 merged sequence pairs were obtained; however, after quality filtering, only 3,203,861 high-quality reads remained that were binned at 97% sequence identity into 680 OTUs. Manual inspection of 127 suspicious OTUs with low identity to fungal ITS revealed many sequences of plant, protest, or bacterial ribosome DNA, so these were excluded from analysis, leaving 2,116,837 reads. Of the 377 fungal samples with ITS amplicons, only 375 ended up returning high-quality data, with read counts ranging from just 1 for barley sand root #1 to 67,329 for coffee seed #1. Fungal ITS read counts averaged about 5,600 per sample; however, without a way to block amplification of non-target reads such as plant ITS sequences (which were bioinformatically removed), these averaged 8,058 per sample; many OTU counts for seed, spermosphere, and shoot samples came out very low. To compare the diversity in different samples using Bray-Curtis dissimilarity, we rarefied the data to 1,000 reads per shoot, 1,000 reads per root, and 2,000 reads per rhizosphere (although samples with low OTU counts were included without rarefaction); OTU counts were transformed to relative proportions, then averaged across repetition. For all other figures and tables, reads from different reps were summed together, then transformed to relative proportion.

Operational taxonomic units sequences and their taxonomy are included as Supplementary Table 3 (bacterial 16S OTU counts/taxonomy) and Supplementary Table 4 (fungal ITS OTU counts/taxonomy). Raw sequencing files were submitted to the NCBI SRA under BioProject PRJNA731997.

### Microbes in Seeds and Spermospheres

All 68 seed and spermosphere samples yielded bacterial and fungal rDNA sequences, which were summed across biological replicates and normalized to proportional abundance (Supplementary Data Sheet 1). Seeds contained an average of 56 bacterial OTUs each and an average Shannon *H* index of 2.2. There was a lower fungal diversity with an average of 14.2 OTU per seeds, with an average Shannon *H* index of 1.4. Spermospheres contained an average of 133 bacterial OTUs each and had an average Shannon *H* index of 2.4. Again, fungal diversity in spermospheres was lower than that for bacteria, with an average of 22.8 OTUs per sample and an average Shannon *H* index of 1.7.

**FIGURE 2:**
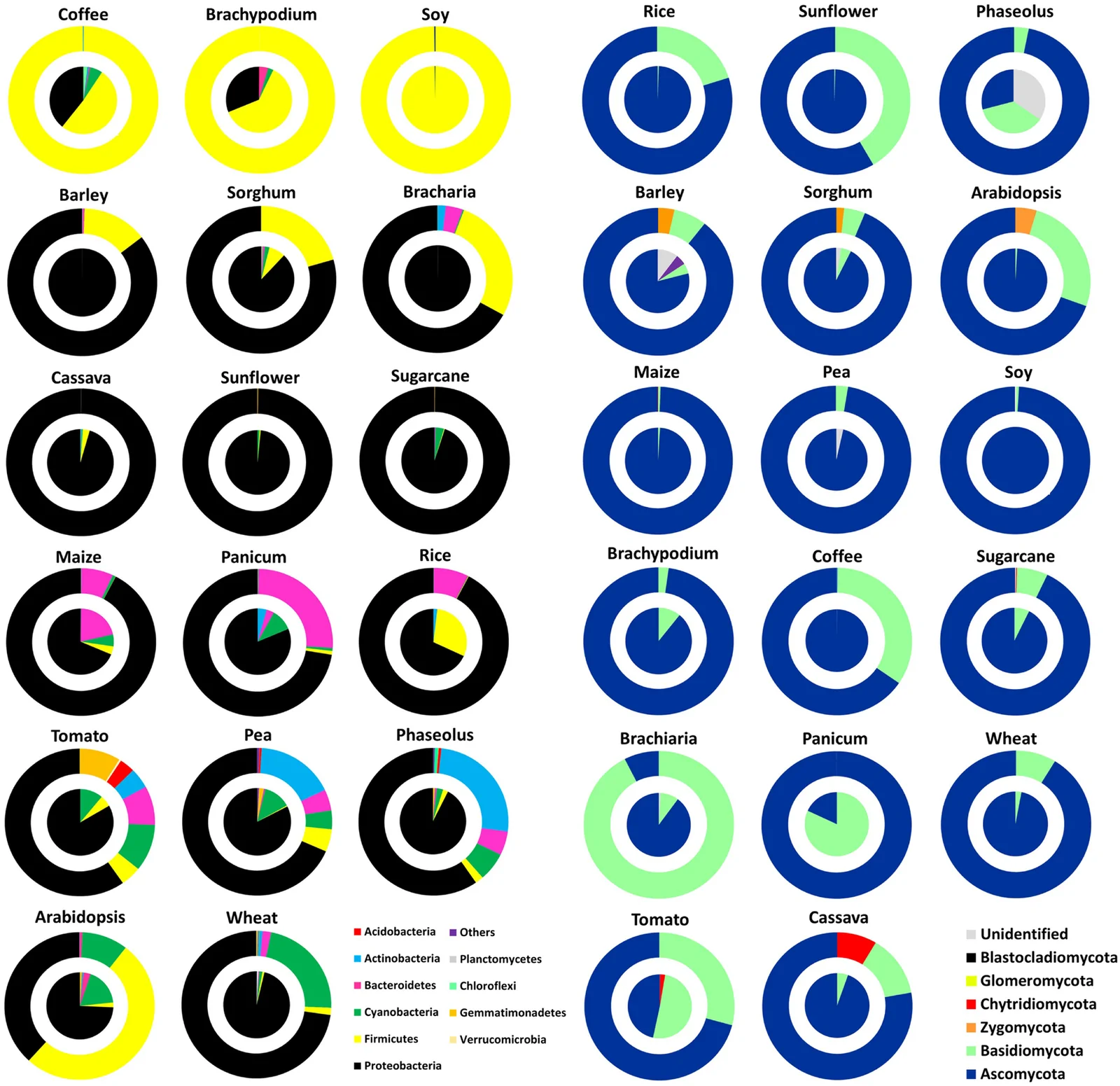
Phylum level classification of bacterial 16S rDNA **(A)** and fungal ITS DNA **(B)** amplified from seeds (pies) and spermospheres (donuts) of the 17 different plant species used in this study. OTUs in each repetition were grouped by sample type and color classified by phylum to yield relative proportion by sample. Seeds and spermospheres were added together and sorted by agglomerative hierarchical clustering using Bray–Curtis dissimilarity of phylum distribution.

A graphical overview of phylum-level microbial taxonomy is displayed for seeds and spermospheres (Figure 2). Nearly all seeds were dominated by OTUs belonging to Proteobacteria and Ascomycetes, with the notable exceptions of coffee, soy, and *Brachypodium*, whose microbiomes were dominated by Firmicutes, while *Panicum*, tomato, and *Phaseolus* seed mycobiomes were made up mostly of Basidiomycetes and other unknown fungi. Bacterial diversity on seed surfaces was greater than that on interiors, but there appeared to be some influence of one to the other. The most obvious examples are bacteria in coffee, soy, and *Brachypodium* spermospheres and seeds that were both dominated by Firmicutes, while both the inside and surface of cassava, sunflower, and sugarcane seeds were nearly all Proteobacteria. The same trend did not appear to hold true for fungal populations on and inside seeds; for example, barley, sorghum, and *Arabidopsis* spermospheres contained zygomycete OTUs that were not detected inside seeds; coffee spermospheres were rich in Basidiomycetes though none were detected inside seeds; Chytridiomycete sequences were found in cassava spermospheres but not inside seeds; and *Panicum* seeds were dominated by Basidiomycetes unlike spermospheres where none were observed.

**FIGURE 3:**
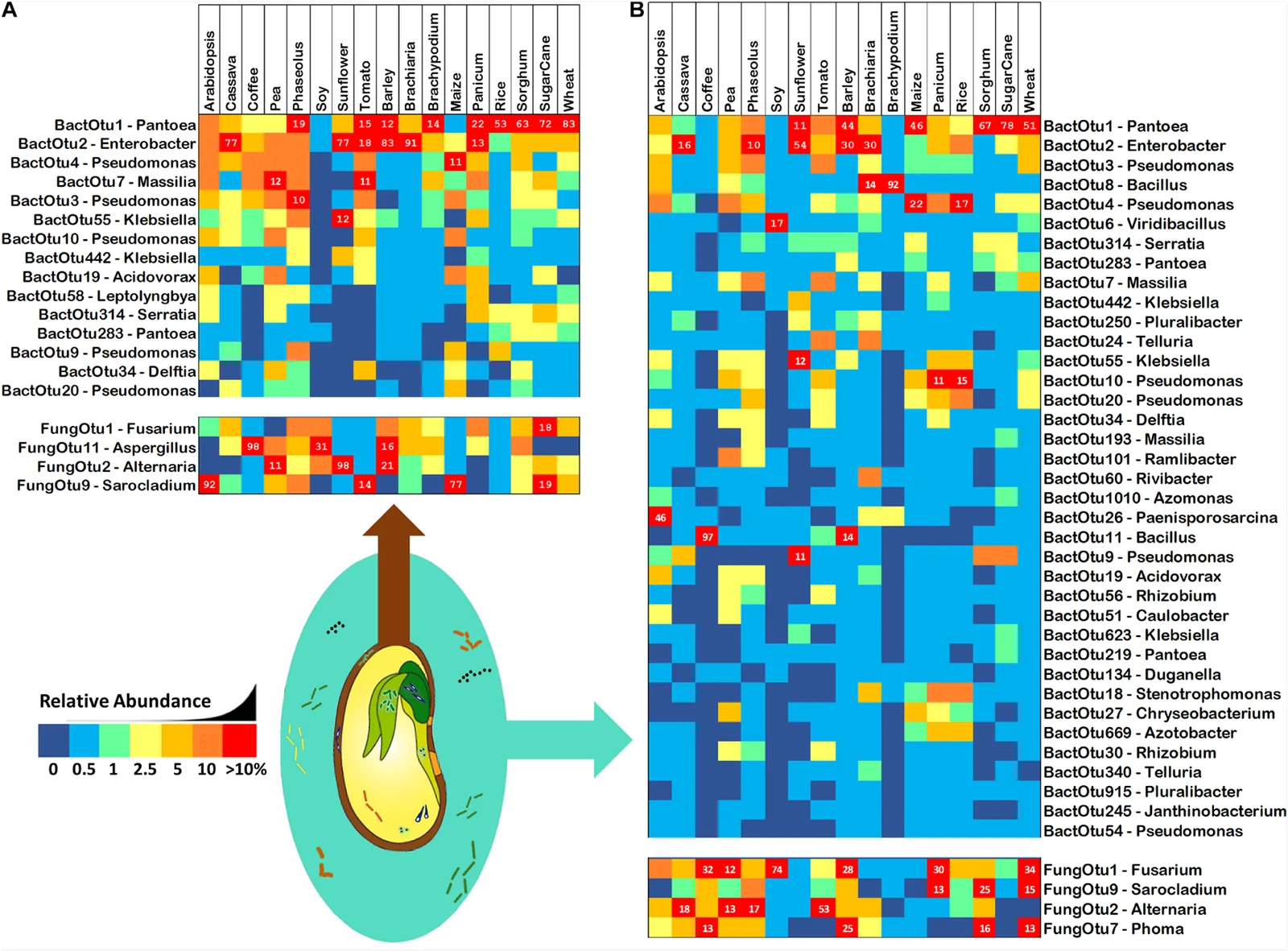
Common bacterial 16S and fungal ITS OTUs of **(A)** seeds and **(B)** spermospheres. Common OTUs were defined as having an occupancy of more than 60% across seeds or spermospheres of the 17 different plant species. Bacterial OTUs are shown in the top blocks, fungal OTUs on the bottom blocks. Next to each OTU ID# is the predicted genus of that sequence. OTU read proportion is represented by color as shown in the legend, with red squares also showing the proportion as a number.

In an attempt to discover common and core microbiomes of angiosperm seeds and spermospheres, Figure 3 shows all OTUs with an occupancy in more than 60% of plant species. There were 15 and 37 bacterial OTUs common to angiosperm seeds and spermospheres, respectively (together totaling 38 OTUs), while fungi were much less common with only 4 OTUs appearing in more than 60% of samples (together totaling 5 OTUs). The only core bacteria in seeds were *Pantoea* (BactOTU1) and *Enterobacter* (BactOTU2), which also appeared in all spermospheres. *Pseudomonas* (BactOTU3) and *Bacillus* (BactOTU8) were also core to spermospheres, as was *Fusarium* (FungOTU1).

### Vertically Transmitted Microbes in Sterile-Grown Shoots, Roots, and Rhizospheres

**FIGURE 4:**
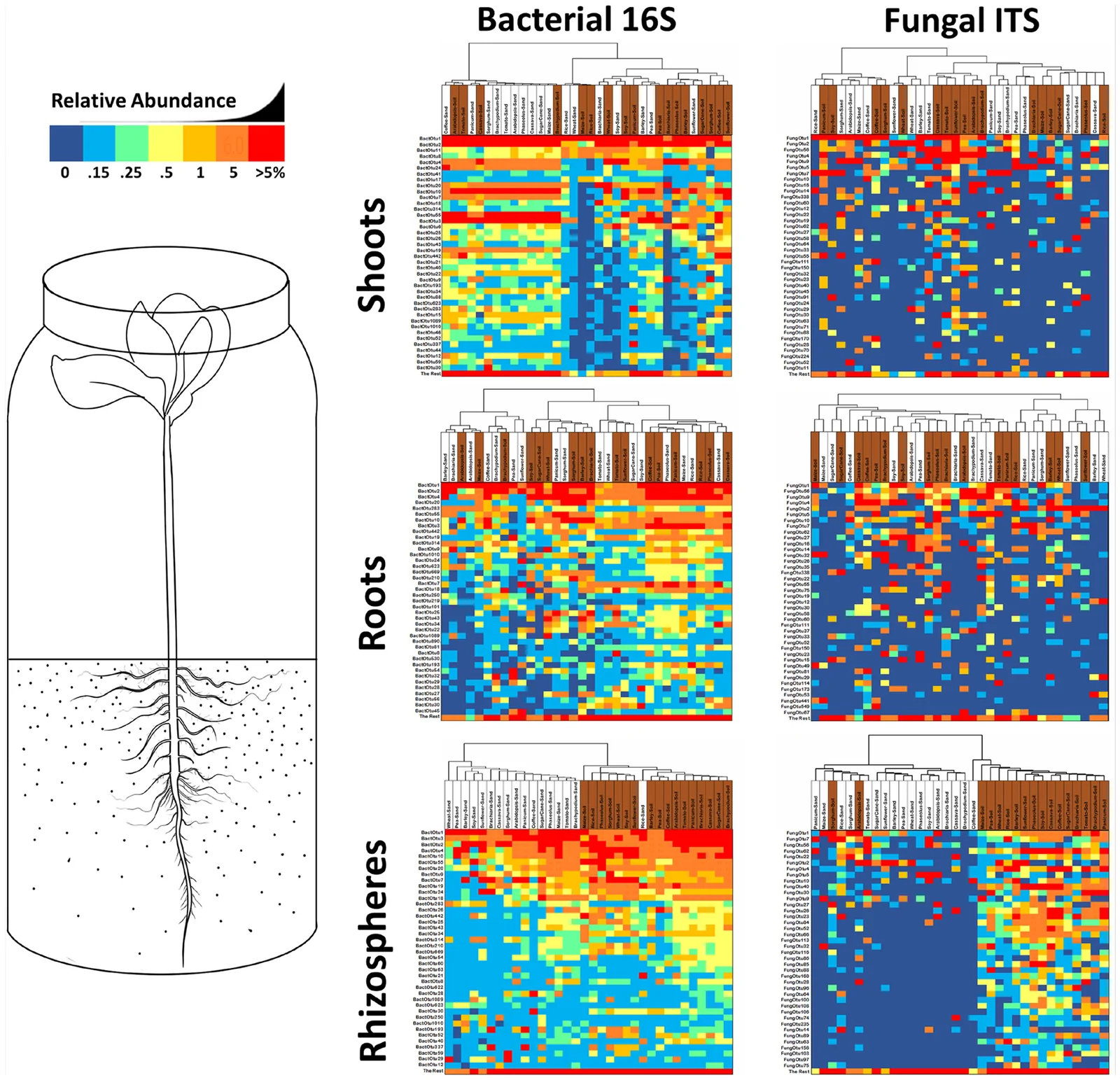
Heatmaps of the 40 most common bacterial 16S rDNA and fungal ITS OTUs derived from Miseq analysis of rhizospheres, roots and shoots from 17 different plant species grown in sealed jars on either sterile sand or field soil. Reads were rarified for each sample (fungi—1000/roots and shoots, 2000/rhizospheres; bacteria—3000/roots, 3500/shoots, 6500/rhizospheres), averaged across repetitions, transformed into relative percentages, then clustered by Bray–Curtis dissimilarity. Samples from sand grown plants are labelled in white, samples from soil grown plants are labelled in brown. Cells are shaded by percentage value with 0% being dark blue, up to 0.1% being light blue, between 0.1–0.25% being green, 0.25–0.5% being light yellow, 0.5-1% being dark yellow, 1-5% being orange and greater than 5% being red.

Seedlings growing on sterile sand inside sealed jars could only receive their microbiomes from seeds. All 306 shoot, root, and rhizosphere samples yielded sequences of bacterial and fungal rDNA. To statistically compare plants by tissue and across species and substrate, samples were rarefied (although samples with low OTU counts were included without rarefaction), normalized to proportional abundance, averaged across reps, and then ordered horizontally by Bray–Curtis dissimilarity. The 40 bacterial 16S and fungal ITS OTUs with highest occupancies are presented in descending order in roots, shoots, and rhizospheres of all 17 plant species grown on both sterile sand and field soil (Figure 4). As evidence of seed transmission, many of the bacterial OTUs appear in all samples regardless of substrate, while only a couple of the highest occupancy fungal OTUs appear across the board. Further evidence of seed transmission could be seen if both sterile sand and soil-grown samples of a particular species group together. For shoots, it is difficult to see whether seed or soil transmission was more important; however, bacteria populations in rice, soy, and pea samples clustered together, while fungal populations in coffee, wheat, tomato, and sugarcane were grouped into the same clade. Inside roots, seed transmission had a clear impact on endosphere microbiomes relative to soil, with 6/17 bacterial samples and 8/17 fungal root samples clustering by plant species. Except for sand and soil-grown fungal populations in sorghum rhizospheres that were put in the same clade, there was no clustering of rhizosphere samples by plant species.

To inventory the microbes in sterile sand-grown plants, OTUs were summed together across the three biological replicates and normalized (Supplementary Data Sheet 2). Rhizospheres contained an average of 252 bacterial OTUs each, roots contained an average of 177 bacterial OTUs each, and shoots contained an average of 255 bacterial OTUs each. There were much lower numbers of fungal OTUs observed in plants growing in sterile sand, with rhizospheres containing an average of 25 fungal OTUs each, roots containing an average of 24 fungal OTUs each, and shoots containing an average of 16 fungal OTUs each. There were 41 core bacterial OTUs that occurred in all rhizospheres of sterile sand-grown plants, 20 in roots, and 56 in shoots. Of these core bacteria, there were 18 that were observed in all three sample types of all plant species, most abundant of which were the previously noted core seed OTUs *Pantoea* (BactOTU1), *Enterobacter* (BactOTU2), and *Pseudomonas* (BactOTU3 or 4) (Table 1). Several other OTUs of *Pseudomonas* (BactOTU9, 10, 20, and 54) appeared in all tissues of all plants, as did *Klebsiella* (BactOTU55 and 623), *Massilia* (BactOTU10), *Acidovorax* (BactOTU19), *Telluria* (BactOTU24), *Stenotrophomonas* (BactOTU18), *Rhizobium* (BactOTU30), *Methylobacterium* (BactOTU28), *Serratia* (BactOTU314), and *Pluralibacter* (BactOTU250). Unlike bacteria, there were no fungal OTUs that were observed in all plant samples grown on sterile sand, although as the most commonly observed, FungOTU1 (*Fusarium proliferatum*) was found in 16/17 of the rhizospheres, 15/17 of the roots, and 15/17 of the shoots. The next most common fungus was FungOTU2 (*Alternaria alternata*), which was observed in 16 rhizospheres, 15 roots, and 13 shoots of sand-grown plants, and then came the basidiomycete *Pseudozyma* (FungOTU56) appearing in 16 rhizospheres, 15 roots, and 14 shoots. The OTUs most commonly observed in sterile-grown plants were also usually the most abundant—BactOTU1, 2, 3, and 4 had average abundances of 22, 11, 13, and 7% in sterile sand-grown plant tissues, respectively, while FungOTU1, 2, and 56 had an average abundances of 12, 14, and 2.1%, respectively.

**TABLE 1:** Twenty bacterial 16S and 10 fungal ITS with the highest occupancy in plants grown on sterile sand, with “common” observations shaded in green and average read percentages above 5 shaded in red.

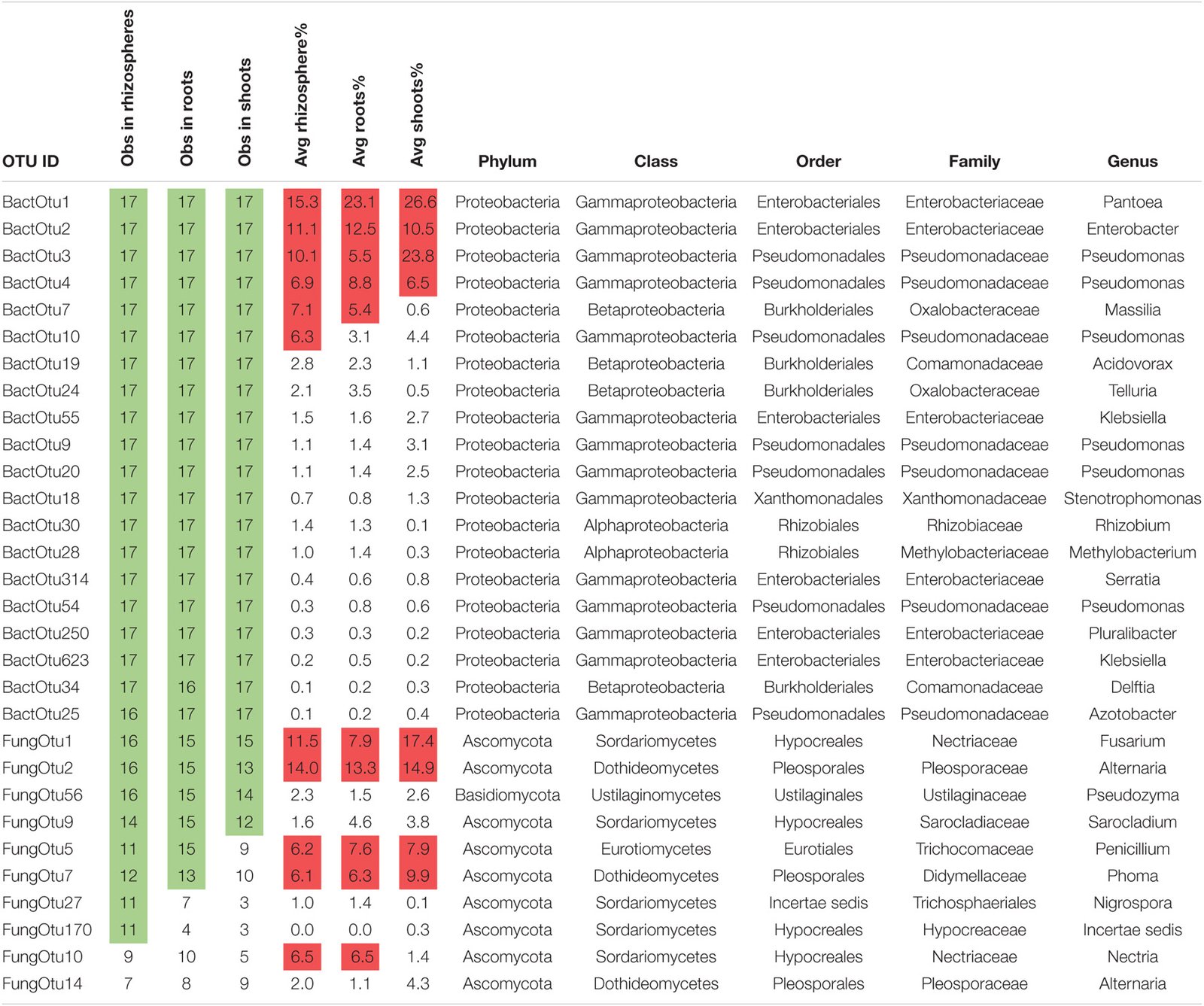

**FIGURE 5:**
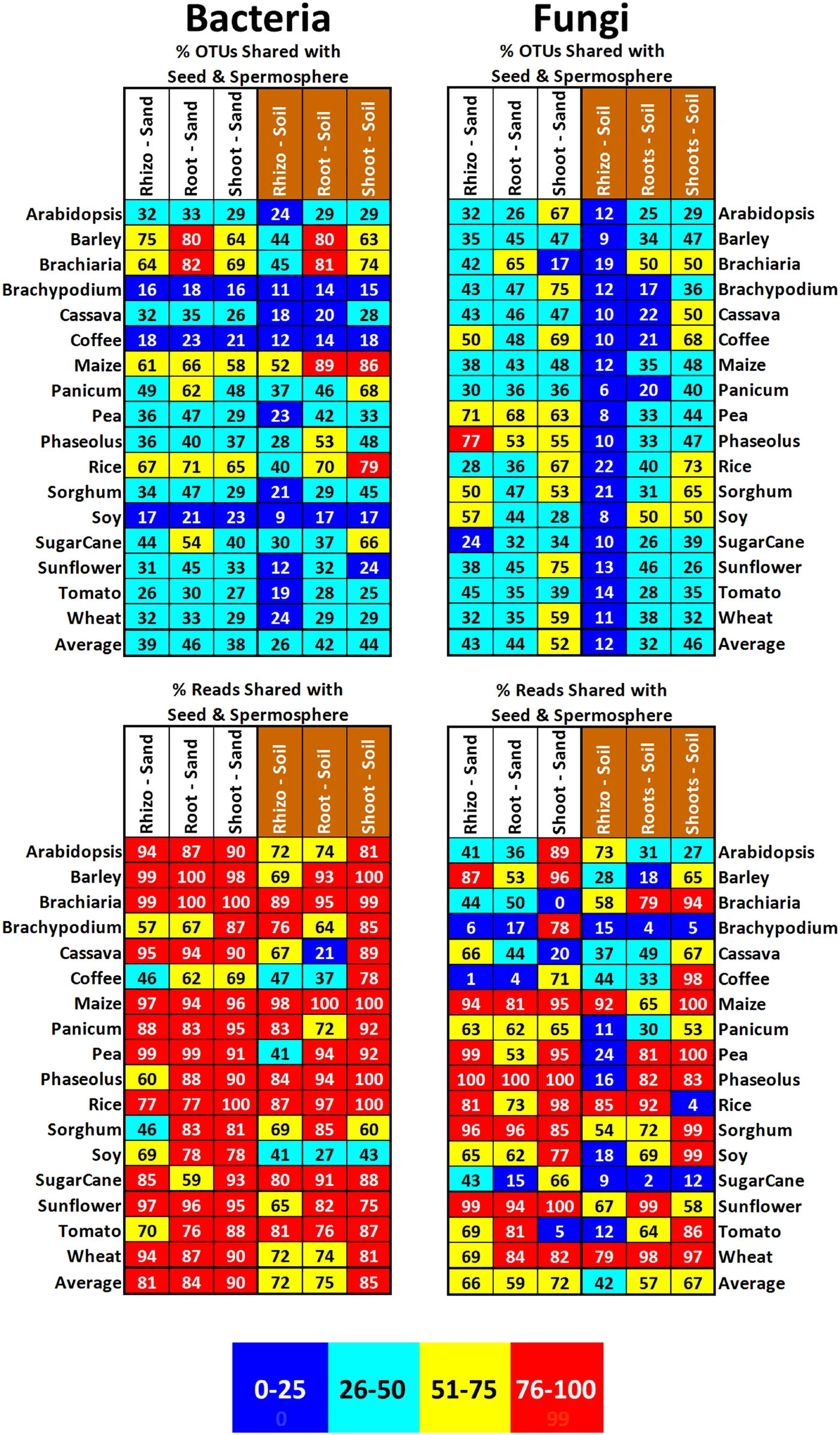
Proportion of OTUs and reads in the shoots, roots, and rhizospheres of different plant species growing either on sterile sand (labeled in white) or field soil (labeled in brown) that were also observed in that species’ corresponding seeds or spermospheres. Cells are shaded to reflect proportion, with 0–25% being blue, 26–50% being green, 51–75% being yellow, and 76–100% being red. An average across all the plant species is shown at the bottom of each column.

Plants growing in sealed jars on sterile sand could only have seeds as their source of microbial inoculum; thus, it was expected that we would find evidence that 100% of all OTU diversity and read abundance came from seeds or spermospheres (Figure 5). By comparing seedling microbiomes to seed/spermosphere microbiomes, vertically transmitted bacteria can only explain a minority of the OTU diversity in shoots, roots, and rhizospheres, representing on average 38, 46, and 39%, respectively. The diversity of vertically transmitted bacterial OTUs in shoots, roots, and rhizospheres of *Brachypodium*, coffee, and soy plants was much lower than that in others, which might be explained by the detection of almost only Firmicutes in their seeds and spermospheres. Calculating average read abundance of vertically transmitted bacteria in shoots, roots, and rhizospheres returned 90, 84, and 81%, respectively, suggesting they predominate over other bacteria from soil or unknown provenance. Some plant samples (*Brachiaria*, barley, and rice shoots; pea rhizospheres and roots) stood out for having 98–100% of their 16S OTU reads deriving from seed-transmitted bacteria.

In plants growing on sterile sand in sealed jars, vertically transmitted fungi were expected to account for 100% of the observable OTU diversity and reads. Surprisingly, there was only evidence for seed transmission to explain about half of the diversity of fungi in shoots, roots, and rhizospheres, or on average 52, 44, and 43%, respectively. This minority of vertically transmitted fungi dominated sterile sand-grown shoot, root, and rhizosphere mycobiomes however, with an average read abundance of 72, 59, and 66%, respectively. Some unusual samples, such as *Brachiaria* and tomato shoots; coffee roots; and *Brachypodium* and coffee rhizospheres, had evidence for less than 6% of their fungal reads coming from seed-transmitted fungi. These anomalies appear to have been caused by disproportionately large proportions of reads being attributed to OTUs that were not detected in the matching seed; for example, 89% of reads belonged to FungOTU45 (*Cryptococcus* sp.) in tomato shoots or 99% of FungOTU10 (*Aspergillus* sp.) in coffee rhizospheres.

### Microbes in Shoots, Roots, and Rhizospheres of Soil-Grown Plants

Seedlings growing on field soil inside sealed jars could receive their microbiomes from either seeds or the substrate they grew on. All 306 samples yielded microbial OTU sequences, which were summed across biological replicates and normalized to proportional abundance (Supplementary Data Sheet 2). Rhizospheres contained an average of 594 bacterial OTUs each, roots an average of 225 bacterial OTUs, and shoots contained an average of 185 bacterial OTUs. Relative to bacteria, there was a lower diversity of fungal OTUs observed in plants growing in soil, with rhizospheres containing an average of 126 fungal OTUs, roots containing an average of 37 fungal OTUs, and shoots containing an average of 24 fungal OTUs.

A total of 10 bacterial OTUs were ubiquitous among all sample types and species growing in soil, the most abundant of which were the previously noted core seed bacteria *Pantoea* (BactOTU1) and *Enterobacter* (BactOTU2) (Table 2). The other 8 OTUs found in all rhizospheres, roots, and shoots included *Pseudomonas* (BactOTU3, 4, 9, and 10), *Klebsiella* (BactOTU55 and 442), *Massilia* (BactOTU7), and *Pantoea* (OTU283). In total, there were 246 bacterial OTUs that occurred in all rhizospheres of soil-grown plants, 18 in roots, and 15 in shoots. Compared to bacteria, there were fewer ubiquitous fungi in soil-grown plants, with only FungOTU1 (*F. proliferatum*) occurring in all rhizospheres, roots, and shoots. *F. solani* (FungOTU4) was observed in all rhizospheres and all shoots, but only 16/17 in roots, while *A. alternata* (FungOTU2) was observed in 13/17, 15/17, 16/17 rhizospheres, roots, and shoots, respectively. FungOTU56 (*Pseudozyma* sp.) was observed in all root samples, but only 14/17 in rhizospheres and 12/17 in shoots. In total, there were 10 fungal OTUs that occurred in all rhizospheres of soil-grown plants, 2 in roots, and 2 in shoots. The OTUs most commonly observed in soil plants were again the most abundant—BactOTU1, 2, 3, and 4 had average abundances of 16, 9, 9, and 7%, while FungOTU1, 4, and 2 had average abundances of 7, 10, and 14%. FungOTU56 was anomalous, having an average abundance of only 1%, which was even lower than that in plants grown on sterile sand.

**TABLE 2:** Twenty bacterial 16S and 10 fungal ITS with the highest occupancy in plants grown on soil, with common observations shaded in green and average read percentages above 5 shaded in red.

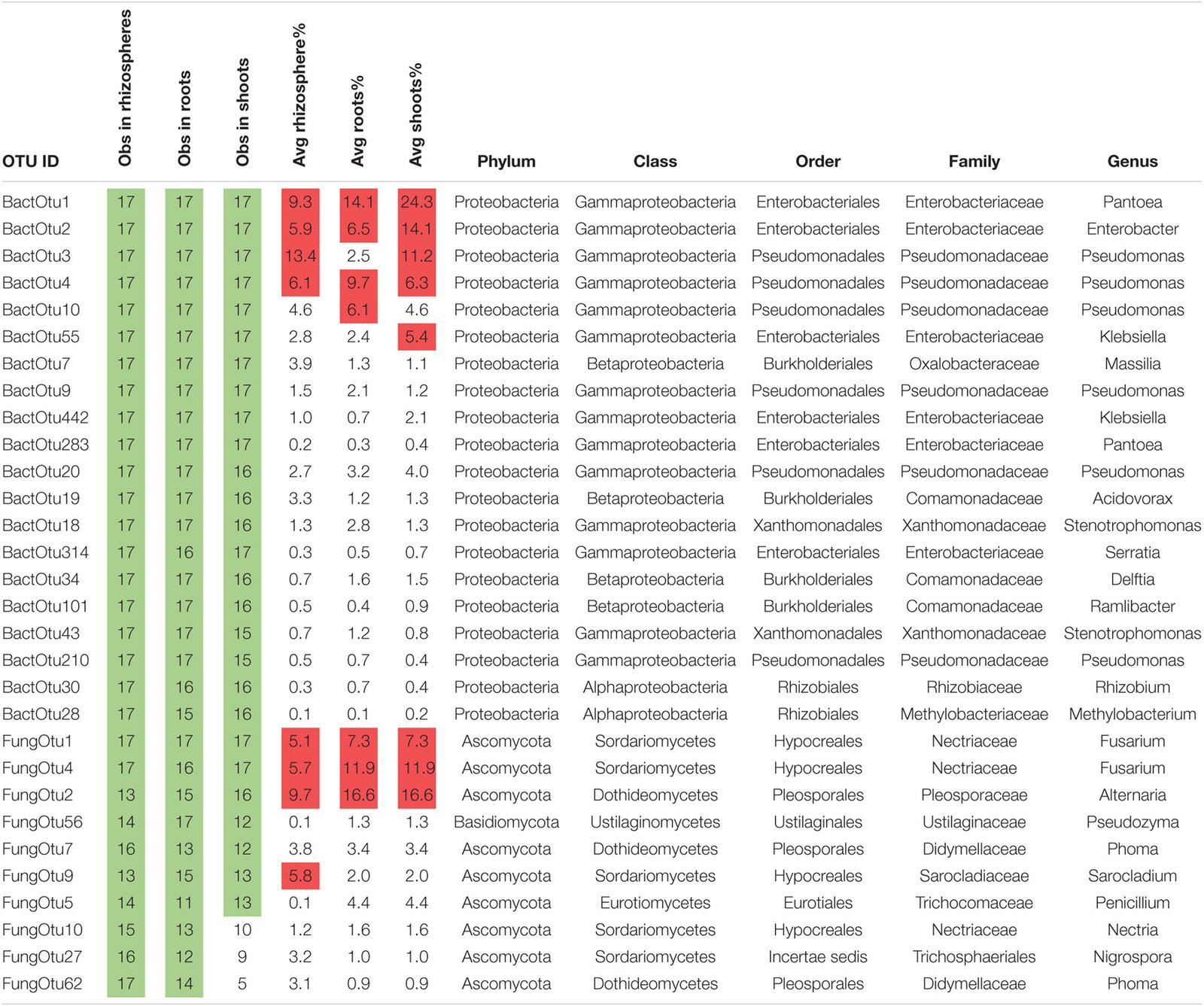

To evaluate the impact of soil on microbiomes of these plants, OTU counts were rarefied (although samples with low OTU counts were included without rarefaction), normalized to percentages, averaged across reps, and then ordered horizontally by Bray–Curtis dissimilarity (Figure 4). Clustering of samples by substrate rather than plant species would indicate soil has a more important role in structuring the microbiome than vertical transmission. Bacterial and fungal populations in rhizospheres show very strong clustering by substrate, with all bacterial samples grouping together into soil and sand clades (except for sand grown rice rhizospheres), while nearly all fungal samples from soil-grown plants (except for soil grown *Arabidopsis* and sorghum) clustered together. Looking at OTU abundance across rhizospheres, it is possible to see that 30 bacterial OTUs occur in all plants on both substrates, but there is no clear trend in abundance fluctuation due to growth on soil. In contrast, fungal OTU rhizosphere abundance is dramatically increased by plant growth on soil, with the majority of fungal OTUs going from absent on sand to significantly abundant on soil. Inside roots, the influence of soil on the diversity or abundance of either bacteria or fungi was reduced in comparison to rhizosphere with only 8/17 bacterial samples and 9/17 fungal samples weakly clustering into various small clades. In shoots, 7/17 soil-grown bacterial samples grouped by substrate in one major clade, and 6/17 soil-grown fungal samples formed one major clade; however, in the rest of the samples, there was no clear soil influence on the diversity or abundance of microbes relative to seed transmission.

Despite growing on non-sterile soil, plants could be acquiring diverse and dominant microbes from their seeds (Figure 5). Comparing OTUs in seeds/spermospheres to those in soil-grown seedlings, vertical transmission of bacteria can only explain a minority of OTU diversity in rhizospheres, roots, and shoots, with an average 26, 42, and 44%, respectively. Again, the diversity of bacterial OTUs in *Brachypodium*, coffee, and soy seedlings was abnormally low, but this was because sequencing of their seeds/spermospheres returned mostly Firmicutes. Vertically transmitted bacteria were ecologically dominant in soil-grown rhizospheres, roots, and shoots, however, with an average read abundance of 72, 75, and 85%, respectively. Of note are some plant samples that were observed to have 97–100% of their reads deriving from seed-transmitted bacteria such as maize (all sample types), barley/*Brachiaria*/*Phaseolus*/rice shoots, and rice roots.

Seed-transmitted fungi (compared to bacteria) appear to be transmitted in similar patterns of diversity and abundance to vegetative tissues of soil-grown plants (Figure 5). Vertically transmitted fungi only explained a minority of the diversity of rhizosphere, root, and shoot populations, representing on average 12, 32, and 46%, respectively. This minority was quite abundant in rhizospheres, roots, and shoots, however, representing an average read abundance of 42, 57, and 67%, respectively. Some soil-grown samples had anomalous OTU read proportions; for example, *Brachypodium* and rice shoots derived less than 5% of their fungal reads from seed transmission, while 97–100% of reads in coffee/maize/pea/sorghum/soy/wheat shoots came from seed-transmitted fungi. The 98–100% of reads in sunflower and wheat root samples come from seed-transmitted fungi, while less than 4% of reads in *Brachypodium* and sugarcane roots do. Fungal diversity inside soil-grown roots and shoots (average of 31 OTUs/sample) was much smaller than that in soil-grown rhizospheres (average of 127 OTUs/sample) and often dominated by one hyperabundant fungus.

**FIGURE 6:**
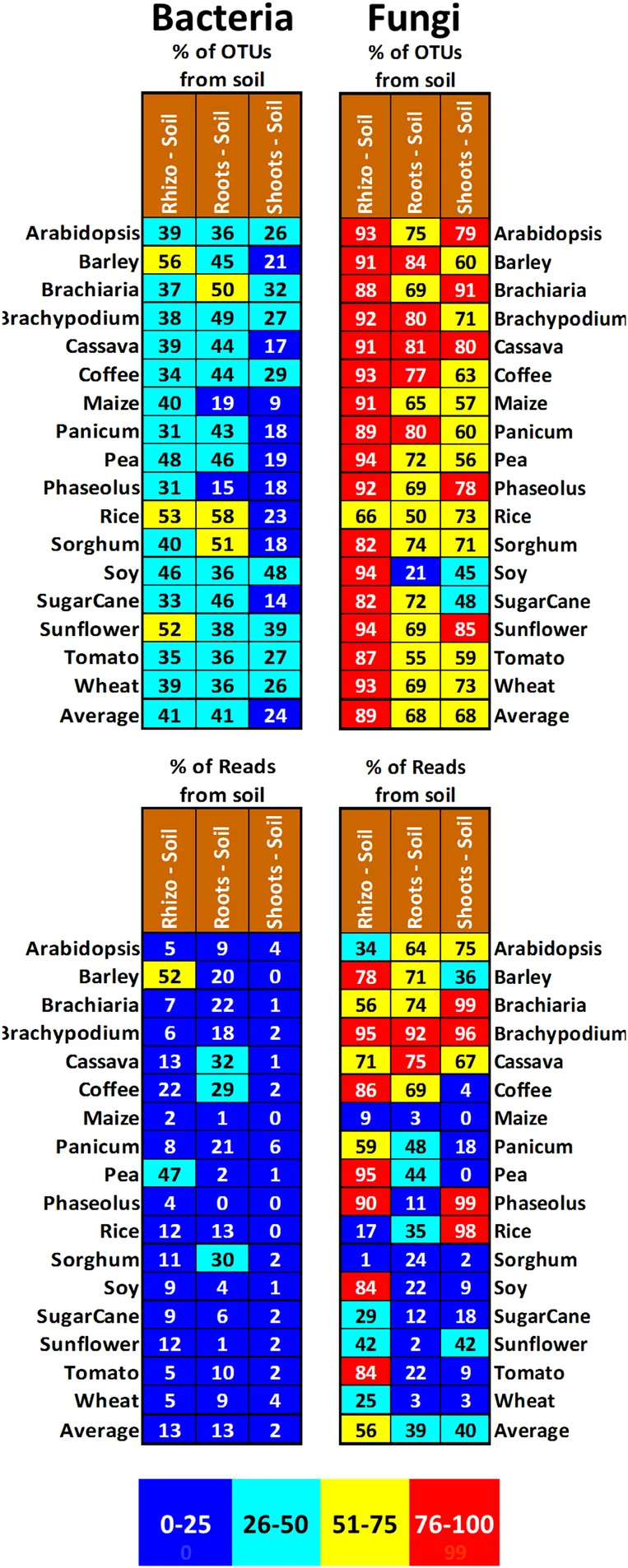
Proportion of OTUs and reads in the shoots, roots, and rhizospheres of different plant species growing on field soil (labeled in brown) that *were not* observed in that species’ corresponding shoots, roots, and rhizospheres from plants grown on sterile sand. Cells are shaded to reflect proportion, with 0–25% being blue, 26–50% being green, 51–75% being yellow, and 76–100% being red. An average across all the plant species is shown at the bottom of each column.

Microbes in soil-grown plants, with no evidence for provenance from seeds, presumably came instead from the substrate. To find the proportion of microbes that might be colonizing plants from the soil, we subtracted all OTUs observed in samples of sterile sand-grown plants from matching sample types of the matching plant species grown on soil (Figure 6). Using this method, on average, 41, 41, and 24% of bacterial OTUs in rhizospheres, roots, and shoots (respectively) could be explained as coming from soil. These soil-transmitted bacteria do not appear to heavily colonize plants, as they only represent on average 13, 13, and 2% of bacterial reads in rhizospheres, roots, and shoots. The influence of soil on fungal diversity is much stronger, with, on average, 89, 68, and 68% of fungal OTUs in rhizospheres, roots, and shoots (respectively) appearing to derive from soil. Surprisingly, these soil-transmitted fungi do not seem to be as dominant as seed-transmitted ones, as they only represent on average 56, 39, and 40% of fungal reads in rhizospheres, roots, and shoots, respectively. There was great variation of soil fungus read abundance between the plant samples; for example, over 90% of all fungal reads in all *Brachypodium* tissues seem to derive from soil, while less than 10% of any fungal reads in all maize tissues seem to derive from soil.

### Common and Core Microbes, Their Abundance, and Their Provenance

**FIGURE 7:**
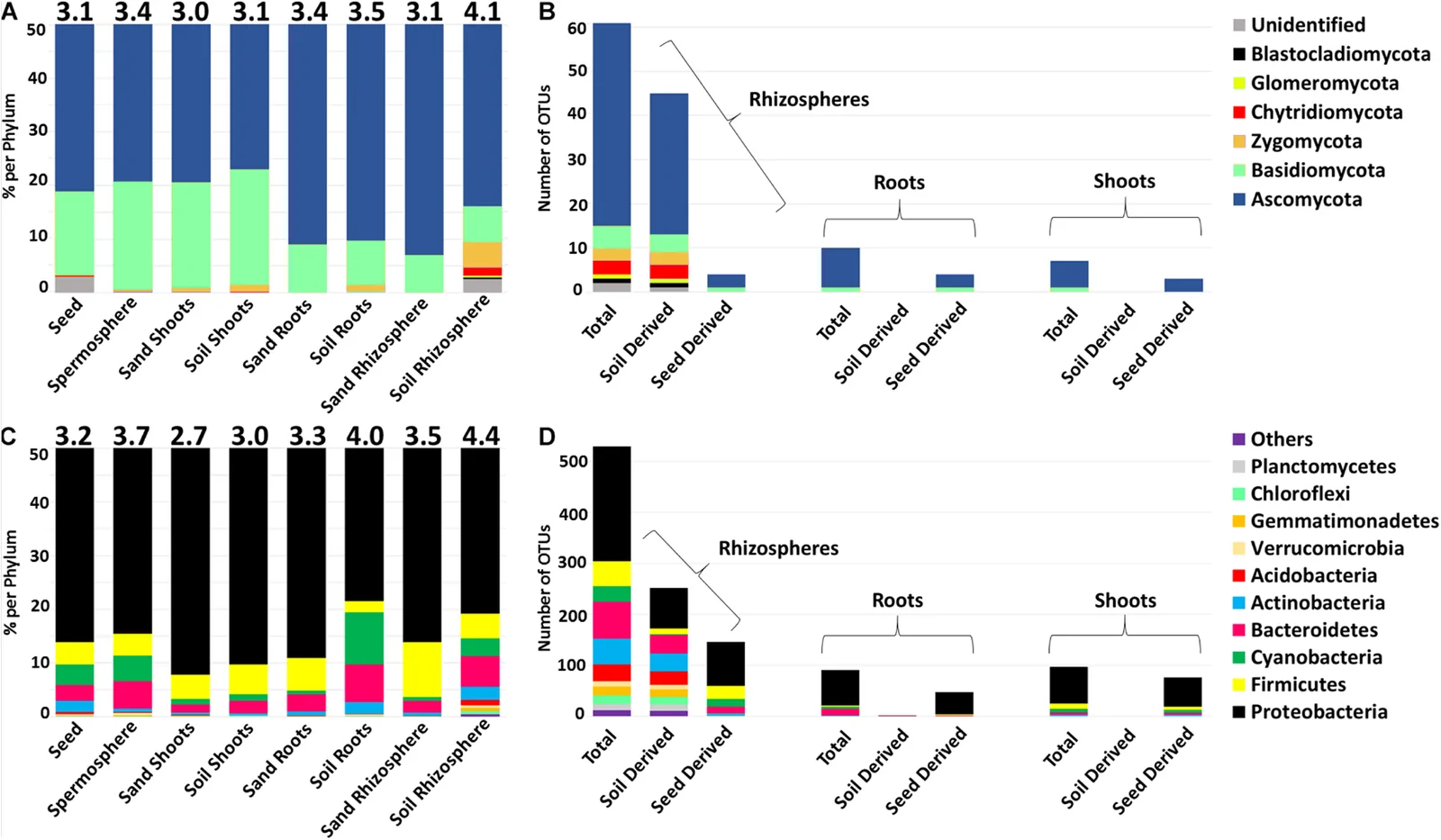
Alpha diversity and phylum-level classification of total **(A,C)** and common **(B,D)** OTUs of fungal ITS DNA and bacterial 16S rDNA amplified from seeds, spermospheres, rhizospheres, roots, and shoots. Alpha diversity was measured using Shannon *H* index and is reported on top of each bar. The proportion of each sample type was calculated by averaging all the samples in each respective group (e.g., average of relative proportions from all seed OTUs). **(B,D)** Total common OTUs were those that were observed in more than 60% of the shoots/roots/rhizospheres of soil-grown plants, while common soil OTUs were observed in more than 60% of soil-grown samples but not in sterile sand-grown samples. Common seed OTUs were those that were observed in both >60% of soil-grown tissues and in >60% of sterile sand-grown tissues. OTUs are classified at the phylum level and shaded according to the legends at the right.

To gain a global view of the microbial diversity in these plants, all samples were averaged together by type, their Shannon *H* diversity was calculated, and all core OTUs were categorized by their phylum-level taxonomy and provenance (Figure 7). Looking at both fungi (Figure 7A) and bacteria (Figure 7C), it was possible to see that microbial diversity of shoots went up slightly when grown on soil (fungi 3.0 to 3.1 and bacteria 2.7 to 3.0) but remained lower than that seen in either seeds or spermospheres. Microbial diversity of roots also went up modestly when grown on soil (fungi 3.4–3.5 and bacteria 3.3–4.0), increasing past that observed in seeds or spermospheres. The most dramatic effect of soil was observed in rhizospheres, where the Shannon *H* diversity index went up by a full point in fungi (3.1–4.1) and 0.9 in bacteria (3.5–4.4). Most of the changes in diversity that were observed in these samples were caused by the appearance or increase in proportion of OTUs from phyla other than Ascomycetes or Proteobacteria.

Rhizospheres had the largest number of OTUs that could be classified as common (occurring in more than 60% of samples) with 61 fungi and 541 bacteria (Figures 7B,D). Not surprisingly, in the rhizosphere, there was evidence that most of these common microbes were transmitted by soil (45 fungi and 263 bacteria), while only a minority had evidence of seed transmission (4 fungi and 146 bacteria). Inside plant tissues, the situation was reversed, as only two out of 91 common bacterial OTUs appeared to be transmitted by soil inside roots, while 47 had evidence of seed transmission. There was a total of 10 common fungal OTUs in roots, none of which came from soil, while four had evidence of seed provenance. In shoots, no common bacterial or fungal OTU had evidence of provenance from soil, but 76 of 97 bacterial OTUs had evidence of seed provenance, while three of seven fungal OTUs appeared to come from seed. Because only seeds or soil were expected to be the source of microbes in this experiment, it was surprising that a substantial number of OTUs did not show evidence of either seed or soil provenance.

Looking at the abundance of common seed and spermosphere microbes in soil-grown plants (Supplementary Data Sheet 2), the 38 common seed/spermosphere bacterial OTUs together were found to represent on average 64% of rhizosphere reads (ranging from 27% in barley to 79% in maize), 67% of root reads (ranging from 14% in *Arabidopsis* to 97% in maize), and 85% of shoot reads (ranging from 40% in sorghum to 100% in maize), while the five common seed/spermosphere fungal OTUs were found to represent on average 25% of rhizosphere reads (ranging from 1% in pea to 88% in maize), 29% of root reads (ranging from 1% in Brachypodium to 95% in wheat), and 40% of shoot reads (ranging from 0.1% in Phaseolus to 99.9% in maize).

In seeds or spermospheres, only *Pantoea* (BactOTU1), *Enterobacter* (BactOTU2), *Pseudomonas* (BactOTU3), *Bacillus* (BactOTU8), and *Fusarium* (FungOTU1) appeared to be part of a core microbiome across the plant species. Because plants grown in sealed jars on sterile sand and irrigated with sterile water could only acquire their microbiomes from inside or on the surface of the seed, we also consider that any of the microbes seen in all rhizospheres, roots, or shoots were both core and seed transmitted. Despite observing few core seed/spermosphere microbes, we observed 41, 20, and 56 core (seed transmitted) bacteria found in all sand-grown rhizospheres, roots, or shoots, respectively, and zero core fungi. Pooling core OTUs from different sample types together, there were thus 63 bacteria and zero fungi that were both core and seed transmitted in sand-grown plants. Of these core seed-transmitted bacteria, there was a total of 18 OTUs that were found in all tissues of sand-grown plants, the most abundant of which were BactOTU1 (*Pantoea*), 2 (*Enterobacter*), 3/4/10 (*Pseudomonas*), 7 (*Massilia*), 19 (*Acidovorax*), 24 (*Telluria*), and 55 (*Klebsiella).* While there was no core fungus in sterile sand-grown plants, FungOTU1 (*Fusarium*) and FungOTU2 (*Alternaria*) were found in nearly all plant tissues and species. When the plants were grown on soil, there were 246, 18, and 15 core bacteria and 10, 2, and 2 core fungi found in all rhizospheres, roots, and shoots, respectively. All 63 core seed-transmitted bacteria observed in sand-grown plants continued to be observed in 100% of either rhizospheres, roots, or shoots of soil-grown plants, and of these, BactOtu1, 2, 3, 4, 7, 10, and 55 continued to be both the most abundant and ubiquitous, found in all rhizosphere, root, and shoot samples. While there had been no core fungus in sterile sand-grown plants, on soil, FungOTU1 (*Fusarium*) was now found in all sample types, while FungOTU56 (*Pseudozyma)* was core to roots and FungOTU4 (*Fusarium*) was found in all shoots.

## Discussion

Seeds are the most direct vehicle that a parent plant might use to transmit microbes to their offspring; however, until recently, experimental science has focused on soil as the major source of the plant microbiome, rarely including sterile substrates as controls and often attempting to sterilize seeds before planting. Within this context, we wanted to characterize and compare the seed-associated microbiomes of 17 of the most important angiosperms including *Arabidopsis*, *Brachypodium*, maize, tomato, rice, and coffee. Does something resembling a core angiosperm seed microbiome exist? Equally important, we wanted to evaluate which of these microbes from natural/unsterilized seed, if any, are transferred to vegetative parts (shoot, root, and rhizosphere) of the plant. Plants were grown in hermetically sealed jars on sterile sand as a way to observe microbiome development in the absence of any other source of inoculum except seeds. Jar-grown plants were also “challenged” with farm soil to see if competition from soil microbes might displace any seed-associated microbes that may have been transmitted to vegetative parts of the plant. High-throughput sequencing was used to identify the taxonomy and relative abundance of bacteria and fungi in these samples, also allowing us to search for patterns of microbiome variation between samples and for evidence of core microbiomes shared between plants and microbial niches. Defining the core microbiomes of model crop plants has been posed as one of the world’s research priorities if we hope to successfully integrate beneficial plant microbiomes into agricultural production (Busby et al., 2017).

### Seed and Spermosphere Microbiomes

Considering that seed surfaces could be an important source of inoculum for vegetative surfaces, while seed endospheres may contribute to the microbiomes of vegetative endospheres, we elected to sample each separately. Every seed surface and interior contained bacterial 16S and fungal ITS sequences (Figures 2, 3); however, with heavy contamination from mitochondria, chloroplast, and plant ribosome sequences and presumably low microbial titers, some seed samples generated very few bacterial or fungal reads (Supplementary Data Sheet 1). Others have speculated that the difficulty in detecting seed-borne microbes results because they are present in small numbers, dormant, and in viable but non-culturable states that resist DNA extraction (Rochefort et al., 2021). Bacteria that we did detect were predominantly Proteobacteria; however, coffee, *Brachypodium*, *Arabidopsis*, and soy seeds or spermospheres were dominated by Firmicutes, which might have meant these would develop distinct Firmicutes-dominated microbiomes when grown into plants (they did not). For fungi, nearly all seeds and spermospheres were dominated by Ascomycetes, except *Panicum* seeds and *Brachiaria* spermospheres, which were richer in Basidiomycetes instead. Attempts to statistically ordinate seed and spermosphere samples by their microbiome diversity and abundance did not result in any apparent pattern, suggesting either we needed more robust data sets or that there is no phylogenetically meaningful structuring of seed microbiomes (data not shown). At the level of OTU, there was substantial commonality (found in >60% of samples) among samples, with 37 common spermosphere bacteria, compared to 15 inside seeds, and four common fungal OTUs in both seeds and spermospheres (Figure 3). Of these common microbes, BactOTU1 (*Pantoea*) and BactOTU2 (*Enterobacter*) were the only ones core to every surface-sterilized seed, but they also occurred in all spermosphere samples, as did BactOTU3 (*Pseudomonas*), BactOTU8 (*Bacillus*), and FungOTU1 (*Fusarium*). Other common and abundant OTUs belonged to the genus *Massilia*, *Klebsiella*, *Alternaria*, and *Sarocladium*.

Reviewed in just the last few years (Truyens et al., 2015), seed microbiomes have been found to contain a diversity of endophytic bacteria. Across 62 species of plants, reported in more than 50 different publications, 155 different bacterial genera primarily from the phyla Proteobacteria, Actinobacteria, Firmicutes, and Bacteroidetes have been detected inside seed tissues, but the dominant genera reported are *Pantoea*, *Enterobacter*, *Pseudomonas*, *Acinetobacter*, and *Sphingomonas* (Hardoim, 2019). Various publications have also reported *Pantoea*, *Enterobacter*, and *Pseudomonas* as core to seeds (Johnston-Monje and Raizada, 2011; Barret et al., 2015; Leff et al., 2017; Yang et al., 2017; Rahman et al., 2018; Eyre et al., 2019; Wassermann et al., 2019b), which coincides with what we observed in this experiment. Fungi living inside seeds can be classified as clavicipitaceous endophytes, which are strictly grass seed-transmitted endosymbionts of the genera *Atkinsonella*, *Balansia*, *Balansiopsis*, *Dussiella*, *Epichloë*, *Myriogenospora*, *Parepichloë*, or as non-clavicipitaceous endophytes, which are mostly Ascomycetes and Basidiomycetes occurring in many different seed types, with the most common genera reported being *Alternaria*, *Fusarium*, *Cladosporium*, *Aspergillus*, *Rhizoctonia*, *Undifilum*, *Chaetomium*, *Colletotrichum*, *Epicoccum*, *Phialophora*, *Tricothecium*, *Cryptococcus*, and *Filobasidium* (Hardoim, 2019). *Alternaria* has been reported as core to some species of plant seeds (Leff et al., 2017; Eyre et al., 2019). Here again, the most common fungal seed endophytes we observed coincided with the non-clavicipitaceous seed endophytes most commonly reported in the literature.

Angiosperm spermospheres are dominated by many of the same microbes that we observed: Proteobacteria (*Agrobacterium*, *Burkholderia*, *Enterobacter*, *Klebsiella*, *Pantoea*, *Pseudomonas*, and *Stenotrophomonas*), Firmicutes (*Bacillus* and *Paenibacillus*), Actinobacteria (*Microbacterium*), Ascomycetes (*Fusarium*, *Penicillium*, *Trichoderma*, *Gliocladium*, *Cylindrocarpon*, and *Cephalosporium*), Basidiomycota (*Rhizoctonia*), and Zygomycota (*Mucor*) (Nelson, 2004, 2018). It has also been shown that lettuce seeds can carry *Olpidium virulentus* (a Chytridiomycete) resting spores externally on the seed coat where they can eventually colonize the spermosphere and begin to infect the developing root (Maccarone, 2013)—we observed Chytridiomycete reads in cassava spermospheres. Similar to our results, some published core spermospheres have been described to contain *Pantoea*, *Pseudomonas*, *Massilia*, *Fusarium*, and/or *Alternaria* (Links et al., 2014; Chen et al., 2016; Klaedtke et al., 2016; Eyre et al., 2019; Chartrel et al., 2021; Moreira et al., 2021).

### Bacteria and Fungi in Shoots

Microbes inside the shoot can influence movement of nutrients and sugars, while on the leaf surface, they can influence gas exchange and harvesting of light. A review of the literature shows that phyllospheres are usually reported to contain Proteobacteria, Firmicutes, Actinobacteria, and Bacteriodetes including the genera *Pseudomonas*, *Bacillus*, *Pantoea*, *Erwinia*, *Sphingomonas*, *Acinetobacter*, *Xanthomonas*, and *Gluconobacter* (Thapa and Prasanna, 2018). Core phyllosphere bacteria in a variety of plants have been observed to include *Methylobacterium*, *Pseudomonas*, *Bacillus*, *Massilia*, *Arthrobacter*, *Rhizobium*, *Pantoea*, and *Sphingomonas* (Delmotte et al., 2009; Rastogi et al., 2012; Horton et al., 2014; Ortega et al., 2016; Wallace et al., 2018; Grady et al., 2019). We observed all of these bacteria in soil-grown shoots of our experiment; however, only *Pantoea*, *Enterobacter, Pseudomonas*, *Klebsiella*, *Massilia*, and *Serratia* were part of a core shoot microbiome across plant species. In literature, the common fungal genera occurring in leaves are *Alternaria*, *Cladosporium*, *Penicillium*, *Acremonium*, *Mucor*, *Cryptococcus*, *Sporobolomyces*, *Rhodotorula*, and *Aspergillus* (Thapa and Prasanna, 2018). Core phyllosphere fungi in a variety of plants have been observed to include *Epicoccum*, *Fusarium*, *Alternaria*, *Cladosporium*, *Cryptococcus*, *Sporobolomyces*, *Udeniomyces*, *Dioszegia*, *Mycosphaerella*, *Plectosphaerella*, *Aureobasideum*, *Neoascochyta*, and *Tetracladium* (Horton et al., 2014; Sapkota et al., 2015; Bowsher et al., 2020). Seed-borne *Fusarium* has been identified as one of the dominant members of the stem endosphere mycobiome of maize (Nebert, 2018). The yeast *Pseudozyma* has been reported as the dominant fungus in and on sugarcane (Nasanit et al., 2015b) and rice leaves as well (Nasanit et al., 2015a; Laur et al., 2018; Wang et al., 2021). The only core fungi in soil-grown shoots in our experiment were both of *Fusarium* (FungOTU1 and 4). We observed *Alternaria* FungOTU2 in 16 of 17 soil-grown shoots and *Pseudozyma* in only 12.

Although the origin of phyllosphere microbes is not well established (Bulgarelli et al., 2013), it is believed that most are environmentally derived from soil, rain, dust, and contact with other organisms, with plant genotype and age playing a major role in shaping and selecting microbes (Whipps et al., 2008; Horton et al., 2014; Wagner et al., 2016). There is also evidence suggesting vertical transmission from seeds to shoots is significant: rice seeds have been shown to populate shoots with bacteria (Hardoim et al., 2012), maize seeds transmit fungi to the leaves (Nebert, 2018), and the growth of oak under axenic conditions suggests that shoots are already heavily colonized by microbes while they exist as embryos inside the seed (Abdelfattah et al., 2021). Observing microbiomes of axenically grown plants gave us another indirect way to see which microbes might be inside seeds, since rare and previously undetectable microbes might have a chance to awaken during seedling germination, allowing them to multiply to levels that are detectable by PCR and sequencing (Shade et al., 2014). We did indeed observe in all plants that their seed can transmit both bacteria and fungi to shoots. Compared to roots and rhizospheres, shoot microbiomes in fact seem to possess the highest level of seed-derived microbes in the plant: about half of bacterial and fungal diversity in shoots seemed to come from seeds, while the majority of reads belonged to these dominant seed-derived microbes.

Ordination of shoot microbiome data did not clearly show clustering by plant species or soil, suggesting neither is a more important source of inoculum for either bacteria or fungi. Comparing soil-grown to sand-grown plants, soil was a very poor source of bacteria for shoots, although for some plants, it did serve to inoculate leaves with diverse or dominant fungi (Figure 6). *Brachiaria*, for example, got 91% of its fungal OTUs from soil, which represented 99% of the reads. Meanwhile, less than 1% of fungal reads in maize and pea shoots came from soil. Because shoots are physically separated from soil (as opposed to roots), perhaps plants have more of a chance to impose tight controls on the number and diversity of microbes that invade their stems and leaves. It may also be that different plants have different ecological strategies, with some practicing more stringent “biotic filtering” (the ability of a plant to restrict which endophytes may enter) than others. Possible examples of strong biotic filtering in shoots, *Bromus tectorum* or maize grown on soils containing significantly different endophytic fungal communities nevertheless develop leaf mycobiomes that are similar (Nebert, 2018; Ricks and Koide, 2019). The lack of clear clustering also leads us to speculate that our experimental setup excluded some other important variables that are important for populating shoot microbiomes, for example exposure to rain (Mechan-Llontop et al., 2021), dust-fall, or surface contact with insects, which have been observed to exert such strong effects on bacterial phyllosphere diversity that tomato leaves were practically identical to synthetic plastic surfaces nearby (Ottesen et al., 2016). The diversity of fungal endophytes in leaves of tropical forest grasses has been found to depend on dispersal limitation (Higgins et al., 2014), but our use of a filtered and homogenized soil as inoculum makes it unlikely that this was a factor in our experiment. Another important variable to consider is that hermetically sealing and growing these plants within glass jars resulted in extremely high humidity and an abnormal atmosphere, which may also have altered microbial diversity and reduced microbial abundance in phyllospheres as has been shown for laboratory- vs. field-grown lettuce (Williams and Marco, 2014).

### Microbial Populations in Roots

Functioning to absorb nutrients and water while secreting biochemicals to manipulate the surrounding microbiology, roots grow into the soil where they have typically been assumed to acquire all their bacterial endophytes (Vandenkoornhuyse et al., 2015). Studies of the root microbiome of *Arabidopsis* (Lundberg et al., 2012), barley (Bulgarelli et al., 2015), rice (Edwards et al., 2015), grape (Zarraonaindia et al., 2015), and sugarcane (Yeoh et al., 2016) have shown that bacterial root endophytes are predominantly Actinobacteria, Bacteroidetes, and Proteobacteria (Bulgarelli et al., 2013). At the level of genus, these bacterial root endophytes include *Acidovorax*, *Agrobacterium*, *Arthrobacter*, *Bacillus*, *Curtobacterium*, *Enterobacter*, *Erwinia*, *Methylobacterium*, *Micrococcus*, *Phyllobacterium*, *Pantoea*, *Pseudomonas*, *Rhizobium*, *Serratia*, *Stenotrophomonas*, *Streptomyces*, and *Xanthomonas* (Hallmann and Berg, 2006). We observed that the dominant/core genera of bacteria in both sterile sand- and soil-grown roots were (in descending order) *Pantoea*, *Enterobacter*, *Pseudomonas*, *Massilia*, *Acidovorax*, *Klebsiella*, and *Stenotrophomonas.* Many other bacteria such as *Rhizobium* and *Methylobacterium* were common, but did not appear in 100% of root samples. *Pantoea*, *Enterobacter*, and/or *Pseudomonas* have been identified as part of a core root microbiome in barley (Yang et al., 2017), coffee (Fulthorpe et al., 2020), tomato (Lee et al., 2019), sugarcane (Yeoh et al., 2016), *Arabidopsis* (Bulgarelli et al., 2012; Lundberg et al., 2012), and diverse seedlings (Barret et al., 2015). *Mas*s*ilia* was reported as a core sugarcane root endophyte (Yeoh et al., 2016), while *Acidovorax* was identified as part of a core root microbiome across 30 species of crop plants (Fitzpatrick et al., 2018). Being legumes, pea, and soy roots were expected to be heavily colonized by rhizobia (BactOTU30), they however represented only less than 0.05% of the reads in either plant, while surprisingly, this OTU made up 21% of the reads in coffee roots growing on sterile sand.

Fungal endophytes of roots are also believed to be soil derived and thus very sensitive to the biogeography of plant growth (Bonito et al., 2014; Bokati et al., 2016; Durán et al., 2018). These communities are usually dominated by Ascomycetes (Dothideomycetes, Sordariomycetes, Leotiomycetes, Eurotiomycetes, and Pezizomycetes), Basidiomycota (Agaricales, Russulales, and Polyporales), and Zygomycota (Porras-Alfaro and Bayman, 2011). Monocots growing in grassland ecosystems have been observed to have root endospheres dominated by Dothideomycetes and specifically *Fusarium* and *Alternaria*, while in forest ecosystems, root endospheres are dominated by Leotiomycetes (Bokati et al., 2016; Jumpponen et al., 2017). The soil-dwelling Chrytidiomycete *Olpidium* has also been observed to intensively infect roots of lettuce (Maccarone, 2013), tomato (Johnston-Monje et al., 2017), melon (Stanghellini et al., 2010), and *Arabidopsis* (Durán et al., 2018). In our experiment, the dominant genera of seed-transmitted fungi in roots grown on sterile sand were *Fusarium*, *Alternaria*, *Pseudozyma*, *Sarocladium*, *Penicillium*, and *Phoma*, which also dominated soil-grown roots, although only *Fusarium* and *Pseudozyma* occurred in all samples. Both *Fusarium* and *Alternaria* have been identified as core root fungi in comparisons of poplar, oak, and pine (Bonito et al., 2014), when studying geographic influence on the *Microthlaspi* root mycoobiome (Glynou et al., 2016); in roots of mandarin orange trees (Sadeghi et al., 2019); and in root endospheres of various wild and domesticated Brassicaceae (Glynou et al., 2018). *Fusarium* has also been identified as a dominant member of the coffee root mycobiome (Fulthorpe et al., 2020), while *Alternaria* was part of a core mycobiome among 28 different germinating seeds (Barret et al., 2015). *Pseudozyma* has been reported as the dominant fungus in and on sugarcane (Nasanit et al., 2015b) and rice leaves where it can protect the plant from pathogens by secretion of antibiotics (Nasanit et al., 2015a; Laur et al., 2018; Wang et al., 2021); however, to our knowledge, it has not been reported as a core member of plant root mycobiomes before.

Many published studies on root microbiology, having attempted to sterilize seeds and forgotten to include a sterile substrate as a negative control, nevertheless conclude that most of the root microbiome derives from soil (Vandenkoornhuyse et al., 2015). For example, a study on the recruitment of *Brassica napus* seedling microbiota, which included no sterile soil treatment and obtained very little sequencing data from seeds as opposed to soil, concluded that most of the seedling microbiome comes from soil or other unknown sources (Rochefort et al., 2021). On the contrary, our results show that seeds of all plants tested are able to transmit microbes to their roots (a core set of seed-transmitted Proteobacteria, *Fusarium*, and *Pseudozyma*), and in most cases, these microbes go on to dominate the endosphere despite being grown in microbe-rich soil. Other publications corroborate the importance of vertical transmission in establishing root microbiomes: we have shown twice before that seed-derived bacteria are the dominant members of juvenile maize root microbiomes (Johnston-Monje et al., 2014, 2016), with similar observations having been made in wheat (Walsh et al., 2021), rice (Hardoim et al., 2012), *Arabidopsis* (Truyens et al., 2016), common bean (López-López et al., 2010), barley (Yang et al., 2017; Rahman et al., 2018), sunflower (Leff et al., 2017), and diverse crops (Barret et al., 2015). It has also been noted that these seed-transmitted microbiomes may change in abundance over time, first increasing during germination (Barret et al., 2015) and later being displaced by soil-derived microbes as plants age (Yang et al., 2017). Fungi can also be transmitted by seeds to roots; for example, tomato roots grown in sterile sand contained *Fusarium*, *Alternaria*, *Penicillium*, *Phoma*, and *Cladosporium* (Johnston-Monje et al., 2017), and both sunflower seeds and young roots were dominated by Pleosporaceae (*Alternaria*), although this changed as plants aged (Leff et al., 2017).

On average, measuring by OTU diversity and abundance, both bacteria and fungi populations in roots were largely seed transmitted; however, there was a dramatic variation between some plants. For example, maize roots grown on soil had 89% of their bacterial OTUs coming from seed, while cassava had only 20%. By read abundance, fungi in sunflower roots were 99% seed transmitted, while sugarcane roots were only 2%. Plant genotype-dependent variations in root microbiomes have been often observed (Bonito et al., 2014; Bouffaud et al., 2014; Schlaeppi et al., 2014; Yeoh et al., 2017; Fitzpatrick et al., 2018; Ricks and Koide, 2019; Wang and Sugiyama, 2020) and are usually explained as variation in the plant’s ability to filter or restrict entry of soil microbes, although they could also reflect variation in seed-transmitted microbial inoculum. Likewise, it has been noted that bacterial endophyte populations vary more by plant compartment than they do by the soil they are grown on (Coleman-Derr et al., 2016; Durán et al., 2018), which, rather than invoking biotic filtering, may be explained if seeds are delivering a consistent bacterial inoculum to the embryo, which then develops differently as it colonizes different organs (Abdelfattah et al., 2021). Indeed, rather than soil serving directly as a source of bacterial inoculum, there is evidence that it is variation in soil characteristics, and in particular pH, that induces the shifts in endophyte population structure, which are often observed in these studies (Hardoim et al., 2012; Barnes et al., 2016). Plant age has been shown to be an important factor in structuring the root microbiome (Wagner et al., 2016), which may also be responsible for some of the variations observed in root microbiomes that were not all sampled at the same age.

### Rhizosphere Microbiomes

The first few millimeters of soil around a root is called the rhizosphere, where robust populations of up to 10^11^ microbial cells per gram live, including over 30,000 prokaryotic species, which help mineralize nutrients or protect against invasion by pathogens (Berendsen et al., 2012). Plants can influence the microbiology of the soil around them through rhizodeposition, where their roots secrete organic acids, phytosiderophores, sugars, vitamins, amino acids, nucleosides, mucilage, and even living root cap border cells (Bulgarelli et al., 2013). It has also been discovered recently that plants can directly inoculate the rhizosphere with bacteria (Johnston-Monje and Raizada, 2011), by sloughing off endophyte-filled root cap border cells (Cope-Selby et al., 2017) or by expulsing microbes out of the swollen ends of root hairs (White et al., 2018). In contrast to these recent discoveries, scientists have traditionally believed that all the rhizosphere microbiome “is recruited from the main reservoir of microorganisms present in soil” (Bakker et al., 2013), with publications on *Arabidopsis* (Lundberg et al., 2012), soy (Liu et al., 2019), rice (Edwards et al., 2015), and maize (Peiffer et al., 2013) rhizospheres reflecting this assumption. A great many publications survey the rhizosphere microbiomes of other plants, including barley (Terrazas et al., 2020), sorghum (Schlemper et al., 2017), coffee (Caldwell et al., 2015), common bean (Pérez-Jaramillo et al., 2019), sunflower (Leff et al., 2017), and pea (Turner et al., 2013). In our previous studies on bacteria in maize (Johnston-Monje et al., 2016) and fungi in tomato (Johnston-Monje et al., 2017), we have corroborated that soil adds significant microbial diversity to the rhizosphere; however, we also found that the most abundant members of the juvenile maize rhizosphere are seed-transmitted bacteria. To our knowledge, no published studies have ever directly addressed the importance of seed transmission to the rhizosphere mycobiome. In this experiment we confirmed that soil contributes to microbial diversity in the rhizosphere, and we also found that the most abundant bacteria and fungi in rhizospheres derive from seeds.

Across all plant rhizospheres grown in sterile sand, we observed 41 different core seed-transmitted bacterial OTUs, to which 205 more were added when grown in soil. Among these core seed-transmitted bacteria, 11 were the most abundant/dominant in soil-grown rhizospheres and included, in descending order: *Pantoea*, *Enterobacter*, *Pseudomonas*, *Klebsiella*, *Massilia*, *Acidovorax*, and *Stenotrophomonas*. *Pseudomonas* is a very common rhizobacteria and, along with *Massilia*, *Acidovorax*, and *Rhizobium*, is a dominant member of core rhizospheres of potato (Pfeiffer et al., 2017), tomato (Lee et al., 2019), lettuce (Schreiter et al., 2014), wheat (Schlatter et al., 2020; Simonin et al., 2020), and maize (Walters et al., 2018). In wheat rhizospheres, it bioaccumulates over years of continuous cropping in a way that is “remarkable in view of the broad range of soil types, climates and agronomic conditions under which wheat is cultivated throughout the world” (Weller et al., 2002), building up to levels that eventually suppress the fungus *Gaeumannomyces tritici*, which causes take-all disease. It is interesting to speculate these biocontrol rhizobacteria actually derive from seeds as we observed in our experiment, rather than soil as has always been assumed. Despite being the most abundant rhizosphere bacteria in our experiment, *Pantoea* has only been reported as core in the wheat rhizosphere (Simonin et al., 2020) and was the second most abundant rhizobacteria we observed previously in juvenile maize rhizospheres (Johnston-Monje et al., 2016). *Enterobacter* has been reported as core for tomato rhizospheres (Lee et al., 2019) and as the keystone species in microbial communities on maize root surfaces (Niu et al., 2017), with the ability to travel through the endosphere, exit the roots, and colonize the surrounding soil (Johnston-Monje and Raizada, 2011).

There were no seed-transmitted fungi that colonized the rhizospheres of all 17 plant species growing on sterile sand; however, *Fusarium*, *Alternaria*, and *Pseudozyma* were present in 16/17. On soil, *Fusarium* and *Phoma* were found in all 17 rhizospheres. Of these, *Fusarium* is the only fungus regularly reported as a core rhizosphere inhabitant, being the dominant fungus on root surfaces of tomato (Lee et al., 2019), wheat (Schlatter et al., 2020; Simonin et al., 2020), maize (Cavaglieri et al., 2009), *Brachypodium* (Kawasaki et al., 2016), and sugar cane (Hamonts et al., 2018). Wheat rhizospheres have also been described to variously have *Phoma* and unidentified Chytridiomycetes as part of their core (Simonin et al., 2020), while in another study, the core wheat rhizosphere had *Alternaria* instead (Schlatter et al., 2020).

Soil significantly increased bacterial diversity in rhizospheres, however, the highest read abundance was of seed-transmitted bacteria (Figure 5). Only an average of 26% of bacterial OTUs came from seeds, but these were responsible for an average of 72% of the reads. These OTUs were mostly Proteobacteria of the genera *Pantoea*, *Enterobacter*, *Pseudomonas*, and *Massilia*, which we observed in seeds or spermospheres and have also been observed associated with a variety of plant seeds (Mundt and Hinkle, 1976; Adams and Kloepper, 2002; Mano et al., 2006; Ferreira et al., 2008; Kaga et al., 2009; Johnston-Monje and Raizada, 2011; Truyens et al., 2013). We have previously observed seeds transmitting dominant bacterial strains (including *Burkholderia*, *Pantoea*, and *Massilia*) into maize rhizospheres (Johnston-Monje et al., 2016). Bacterial endophytes tagged with GFP such as *Enterobacter* from maize seeds (Johnston-Monje and Raizada, 2011) and *Pantoea* from eucalyptus seeds (Ferreira et al., 2008) have also been shown to be able to colonize the endosphere, exit the root, and colonize the rhizosphere. Rice and millet seed endophytes have been later observed in rhizosphere soil (Hardoim et al., 2012; Verma and White, 2018), and seed-transmitted bacteria have been observed colonizing rhizospheres as they emerge from inside sloughed off *Miscanthus* root border cells (Cope-Selby, 2013). Seed-transmitted microbes colonizing the rhizosphere would be guaranteed first access to that habitat, perhaps creating a founder effect, blocking later colonization by less-adapted soil microbes or pathogens (Bacilio-Jiménez et al., 2001; Barka et al., 2002). Seed-transmitted rhizosphere microbes might also play an important role in plant nutrition, for example in the cardon cactus, where they help to mineralize the surrounding rock for nutrient absorption by roots (Puente et al., 2009), get intracellularly taken up by the root, and digested by the plant in a process called rhizophagy (White et al., 2018), or in grasses where dying bacteria release organic nitrogen for absorption by the plant (White et al., 2015).

Compared to bacteria, there was less seed-transmitted fungal diversity in rhizospheres, with only an average of 12%; however, these OTUs tended to become abundant, representing an average of 42% of the reads. Abundance of seed-transmitted fungal reads varied widely and unexplainably by plant, for example with sugarcane having only 9% while maize had 92%. Of these seed-transmitted rhizospheric fungi, FungOTU1 (*Fusarium*) was the most abundant, occurring in all soil-grown rhizospheres, as it did in all spermospheres. We have previously observed that tomato rhizospheres are dominated by seed-transmitted *Fusarium* (Johnston-Monje et al., 2017), but we are not aware of other examples of seed transmitted rhizospheric fungi. Seeking to protect against soil-transmitted seedling pathogens from the genera *Fusarium*, *Rhizoctonia*, *Colletotrichum*, *Cylindrocarpon*, *Pyrenophora*, and *Cochliobolus*, the plant agriculture industry commonly coats seed with fungicides but has not explicitly paid attention to the possibility of a seed-transmitted rhizosphere mycobiome (Nelson, 2018), which might make this the first publication explicitly documenting this phenomenon.

### Some Caveats

Microbial detection in seeds while using PCR to amplify 16S or ITS sequences, followed by Illumina sequencing has been shown to miss as much as 50% of the sequence diversity in an environmental sample (Hong et al., 2009). Shifting patterns of microbiome diversity can be also be obscured when relying solely on the sequencing of 16S or ITS rDNA (as we have) (O’Donnell et al., 2015; Peay et al., 2016; Baltrus, 2020). For example, the frequent crop pathogen *Fusarium oxysporum* comprises a large complex of cryptic species with more than 120 different *formae speciales*, but all sharing the same ITS sequence (Michielse and Rep, 2009). Likewise, three different strains of *Pantoea ananatis* isolated from maize seeds had small but significant differences in their genomes and contrasting effects on plant growth despite sharing identical 16S rDNA (Sheibani-Tezerji et al., 2015).

Microbiomes of axenically grown plants should be 100% seed transmitted; however, this was not the case for any sample, suggesting a problem with capturing the full diversity in seeds and spermospheres. For example, soy seeds were detected to be 99% Firmicutes by abundance containing no *Pseudomonas*, however, when grown on sterile sand, they developed microbiomes similar to most other plants with all the same dominant bacteria. It may be that many seed-associated microbes are exceedingly rare and difficult to detect when they enter viable but non-culturable states such as resting spores that resist DNA extraction (Pollock et al., 2018). PCR of target amplicons may also be a limitation, as it is thought to be limited to detecting the top 99% most abundant sequences in a population (Smalla, 2003). Primer bias also makes it impossible to amplify all the microbial sequence diversity in an environmental sample (Hong et al., 2009), or perhaps some microbial diversity was missed due to stochastic effects of seed selection for sequencing vs. germination. Ironically, sequencing microbiomes of axenically grown vegetative plants may be a better way to observe seed microbes than directly sequencing seeds, since “conditionally rare” and undetectable microbes may get a chance to awaken to more favorable conditions as seeds germinate and grow (Shade et al., 2014).

Another major caveat concerns the nature of the experimental setup, in that growing plants in hermetically sealed jars for a short period of time is not natural, though it did theoretically allow us to control all possible sources of microbial inoculum. These are juvenile plants, which may not yet have developed microbiomes corresponding to mature plants. The abundance of seed-transmitted bacteria we have observed in these young roots, shoots, and rhizospheres may be exaggerated because they have not yet had a chance to be more heavily colonized during passage through soil (Inceoğlu et al., 2011) or exposure to dust-fall or rain (Williams and Marco, 2014). Older and larger plants growing under natural conditions would also have more time to interact with other organisms such as nematodes and insects, which may vector microbes onto the plant and reduce the dominance of seed-transmitted bacteria as they are eaten and killed or displaced. Without possibility for gas exchange, air chemistry, and humidity in these jars was far from what these plants would encounter growing in a farmer’s field, a fact that has been shown to alter microbial diversity and reduce microbial abundance in lettuce phyllospheres (Williams and Marco, 2014).

## Conclusion

This experiment aimed to document the bacterial and fungal diversity in and on seeds of a panel of import plants and observe in a microbially controlled environment, how much of the seed microbiome goes on to form the plant microbiome. Seeds and spermospheres of all 17 plant species contained microbes, mostly Proteobacteria and Ascomycetes. Rhizospheres, roots, and shoots of all 17 plants grown on sterile sand also developed bacterial and fungal populations, showing that seeds are able to transmit complex microbial populations to their seedlings. All of the 63 core seed-transmitted bacteria observed in sterile sand-grown plants were also found in field soil-grown plants, and a subset of seven of these (1 *Pantoea*, 1 *Enterobacter*, 3 *Pseudomonas*, 1 *Klebsiella*, and 1 *Massilia*) were the dominant microbiome members on both types of substrate. There was no core seed-transmitted fungus; however, by tracing the fate of vertically transmitted fungi in individual plant species, it seems that some mycobiomes are also dominated by seed-transmitted fungi, especially *Fusarium* and *Alternaria*. Soil served as a minor source of bacterial diversity to plants, but a major source of diversity for fungi. The most abundant bacteria and fungi in these jar-grown seedlings came from their seeds, not the soil. Future experiments culturing these common and core microbes, cross inoculating them among plant species, and comparing their genetics and physiology may help us understand why they occur so frequently in plant seeds and how they have benefited angiosperm plant physiology over evolutionary time.

## Data Availability Statement

The bacterial and fungal sequence data generated in this study using MiSeq have been deposited and are available in the NCBI Sequence Read Archive (SRA) under BioProject PRJNA731997 and are also provided as Supplementary Material (annotated sequences and OTU counts) in this publication.

## Author Contributions

DJ-M conceived of and designed the study, collected the all materials, performed the all wet lab experiments, conducted the all bioinformatics and statistics, and wrote the manuscript. JG aided in many aspects of the molecular biology lab work. LL-L funded the study and hosted DJ-M in his lab at CIAT. All the authors contributed to the article and approved the submitted version.

## Conflict of Interest

The authors declare that the research was conducted in the absence of any commercial or financial relationships that could be construed as a potential conflict of interest.

## Publisher’s Note

All claims expressed in this article are solely those of the authors and do not necessarily represent those of their affiliated organizations, or those of the publisher, the editors and the reviewers. Any product that may be evaluated in this article, or claim that may be made by its manufacturer, is not guaranteed or endorsed by the publisher.

## Footnotes

1
www.drive5.com

2
https://folk.uio.no/ohammer/past/

## References

[B1] Abdelfattah A. Wisniewski M. Schena L. Tack A. J. M. (2021). Experimental evidence of microbial inheritance in plants and transmission routes from seed to phyllosphere and root. *Environ. Microbiol.* 23 2199–2214. 10.1111/1462-2920.15392 33427409

[B2] Adams P. D. Kloepper J. W. (2002). Effect of host genotype on indigenous bacterial endophytes of cotton (*Gossypium hirsutum L.*). *Plant Soil* 240 181–189. 10.1023/A:1015840224564

[B3] Afzal I. Shinwari Z. K. Sikandar S. Shahzad S. (2019). Plant beneficial endophytic bacteria: mechanisms, diversity, host range and genetic determinants. *Microbiol. Res.* 221 36–49. 30825940 10.1016/j.micres.2019.02.001

[B4] Allard S. M. Ottesen A. R. Brown E. W. Micallef S. A. (2018). Insect exclusion limits variation in bacterial microbiomes of tomato flowers and fruit. *J. Appl. Microbiol.* 125 1749–1760. 10.1111/jam.14087 30146755

[B5] Arif I. Batool M. Schenk P. M. (2020). Plant microbiome engineering: expected benefits for improved crop growth and resilience. *Trends Biotechnol.* 38 1385–1396. 10.1016/j.tibtech.2020.04.015 32451122

[B6] Bacilio-Jiménez M. Aguilar-Flores S. del Valle M. V. Pérez A. Zepeda A. Zenteno E. (2001). Endophytic bacteria in rice seeds inhibit early colonization of roots by *Azospirillum brasilense*. *Soil Biol. Biochem.* 33 167–172.

[B7] Bakker P. Berendsen R. Doornbos R. Wintermans P. Pieterse C. (2013). The rhizosphere revisited: root microbiomics. *Front. Plant Sci.* 4:165. 10.3389/fpls.2013.00165 23755059 PMC3667247

[B8] Baldani J. I. Baldani V. L. (2005). History on the biological nitrogen fixation research in graminaceous plants: special emphasis on the Brazilian experience. *An. Acad. Bras. Cienc.* 77 549–579. 16127558 10.1590/s0001-37652005000300014

[B9] Baltrus D. A. (2020). Bacterial dispersal and biogeography as underappreciated influences on phytobiomes. *Curr. Opin. Plant Biol.* 56 37–46. 10.1016/j.pbi.2020.02.010 32278259

[B10] Barka E. A. Gognies S. Nowak J. Audran J.-C. Belarbi A. (2002). Inhibitory effect of endophyte bacteria on Botrytis cinerea and its influence to promote the grapevine growth. *Biol. Control* 24 135–142.

[B11] Barnes C. J. Maldonado C. Frøslev T. G. Antonelli A. Rønsted N. (2016). Unexpectedly high beta-diversity of root-associated fungal communities in the bolivian andes. *Front. Microbiol.* 7:1377. 10.3389/fmicb.2016.01377 27630629 PMC5006319

[B12] Barret M. Briand M. Bonneau S. Préveaux A. Valière S. Bouchez O. (2015). Emergence shapes the structure of the seed microbiota. *Appl. Environ. Microbiol.* 81 1257–1266. 10.1128/aem.03722-14 25501471 PMC4309697

[B13] Berendsen R. L. Pieterse C. M. Bakker P. A. (2012). The rhizosphere microbiome and plant health. *Trends Plant sci.* 17 478–486. 22564542 10.1016/j.tplants.2012.04.001

[B14] Berg G. Raaijmakers J. M. (2018). Saving seed microbiomes. *ISME J.* 12 1167–1170. 10.1038/s41396-017-0028-2 29335636 PMC5931960

[B15] Berg G. Rybakova D. Grube M. Köberl M. (2016). The plant microbiome explored: implications for experimental botany. *J. Exp. Bot.* 67 995–1002. 26547794 10.1093/jxb/erv466 PMC5395086

[B16] Bokati D. Herrera J. Poudel R. (2016). Soil influences colonization of root-associated fungal endophyte communities of maize, wheat, and their progenitors. *J. Mycol.* 2016:8062073.

[B17] Bonito G. Reynolds H. Robeson M. S. I. I. Nelson J. Hodkinson B. P. Tuskan G. (2014). Plant host and soil origin influence fungal and bacterial assemblages in the roots of woody plants. *Mol. Ecol.* 23 3356–3370. 10.1111/mec.12821 24894495

[B18] Bouffaud M.-L. Poirier M.-A. Muller D. Moënne-Loccoz Y. (2014). Root microbiome relates to plant host evolution in maize and other Poaceae. *Environ. Microbiol.* 16 2804–2814. 10.1111/1462-2920.12442 24588973

[B19] Bowsher A. W. Benucci G. M. N. Bonito G. Shade A. (2020). Seasonal dynamics of core fungi in the switchgrass phyllosphere, and co-occurrence with leaf bacteria. *Phytobio. J.* 5 60–68. 10.1094/pbiomes-07-20-0051-r

[B20] Brachi B. Filiault D. Darme P. Le Mentec M. Kerdaffrec E. Rabanal F. (2017). Plant genes influence microbial hubs that shape beneficial leaf communities. *Biorxiv* [Preprint] 10.1101/181198 Biorxiv: 181198,

[B21] Bulgarelli D. Garrido-Oter R. Münch P. C. Weiman A. Dröge J. Pan Y. (2015). Structure and function of the bacterial root microbiota in wild and domesticated barley. *Cell Host Microbe* 17 392–403. 25732064 10.1016/j.chom.2015.01.011 PMC4362959

[B22] Bulgarelli D. Rott M. Schlaeppi K. van Themaat E. V. L. Ahmadinejad N. Assenza F. (2012). Revealing structure and assembly cues for Arabidopsis root-inhabiting bacterial microbiota. *Nature* 488 91–95. 22859207 10.1038/nature11336

[B23] Bulgarelli D. Schlaeppi K. Spaepen S. Van Themaat E. V. L. Schulze-Lefert P. (2013). Structure and functions of the bacterial microbiota of plants. *Annu. Rev. Plant Biol.* 64 807–838. 23373698 10.1146/annurev-arplant-050312-120106

[B24] Busby P. E. Soman C. Wagner M. R. Friesen M. L. Kremer J. Bennett A. (2017). Research priorities for harnessing plant microbiomes in sustainable agriculture. *PLoS Biol.* 15:e2001793. 10.1371/journal.pbio.2001793 28350798 PMC5370116

[B25] Caldwell A. C. Silva L. C. F. da Silva C. C. Ouverney C. C. (2015). Prokaryotic diversity in the rhizosphere of organic, intensive, and transitional coffee farms in Brazil. *PLoS One* 10:e0106355. 10.1371/journal.pone.0106355 26083033 PMC4471275

[B26] Cavaglieri L. Orlando J. Etcheverry M. (2009). Rhizosphere microbial community structure at different maize plant growth stages and root locations. *Microbiol. Res.* 164 391–399. 17524636 10.1016/j.micres.2007.03.006

[B27] Chartrel V. Dugat-Bony E. Sarthou A.-S. Huchette S. Bonnarme P. Irlinger F. (2021). The microbial community associated with pea seeds (*Pisum sativum*) of different geographical origins. *Plant Soil* 462 405–427. 10.1007/s11104-021-04856-6

[B28] Chaw S. M. Chang C. C. Chen H. L. Li W. H. (2004). Dating the monocot-dicot divergence and the origin of core eudicots using whole chloroplast genomes. *J. Mol. Evol.* 58 424–441. 10.1007/s00239-003-2564-9 15114421

[B29] Chen W. Turkington T. K. Lévesque C. A. Bamforth J. M. Patrick S. K. Lewis C. T. (2016). Geography and agronomical practices drive diversification of the epiphytic mycoflora associated with barley and its malt end product in western Canada. *Agric. Ecosyst. Environ.* 226 43–55. 10.1016/j.agee.2016.03.030

[B30] Cole J. R. Wang Q. Fish J. A. Chai B. McGarrell D. M. Sun Y. (2014). Ribosomal database project: data and tools for high throughput rRNA analysis. *Nucleic Acids Res.* 42 D633–D642. 24288368 10.1093/nar/gkt1244 PMC3965039

[B31] Coleman-Derr D. Desgarennes D. Fonseca-Garcia C. Gross S. Clingenpeel S. Woyke T. (2016). Plant compartment and biogeography affect microbiome composition in cultivated and native Agave species. *New Phytol.* 209 798–811. 10.1111/nph.13697 26467257 PMC5057366

[B32] Compant S. Samad A. Faist H. Sessitsch A. (2019). A review on the plant microbiome: Ecology, functions, and emerging trends in microbial application. *J. Adv. Res.* 19 29–37. 10.1016/j.jare.2019.03.004 31341667 PMC6630030

[B33] Cope-Selby N. (2013). *Diversity and Characterization of Bacteral Endophytes in the C4 Energy Crop Miscanthus.* Ph.D. thesis. Wales: Aberystwyth University.

[B34] Cope-Selby N. Cookson A. Squance M. Donnison I. Flavell R. Farrar K. (2017). Endophytic bacteria in Miscanthus seed: implications for germination, vertical inheritance of endophytes, plant evolution and breeding. *GCB Bioenergy* 9 57–77. 10.1111/gcbb.12364

[B35] Delmotte N. Knief C. Chaffron S. Innerebner G. Roschitzki B. Schlapbach R. (2009). Community proteogenomics reveals insights into the physiology of phyllosphere bacteria. *Proc. Natl. Acad. Sci.U.S.A.* 106 16428–16433. 10.1073/pnas.0905240106 19805315 PMC2738620

[B36] Dent D. Patel D. Devine G. (2017). *Novel Microorganisms and Their Use in Agriculture-WO2017017440A1L.* Mumbai: Greaves.

[B37] Durán P. Thiergart T. Garrido-Oter R. Agler M. Kemen E. Schulze-Lefert P. (2018). Microbial interkingdom interactions in roots promote Arabidopsis survival. *Cell* 175 973–983. 30388454 10.1016/j.cell.2018.10.020 PMC6218654

[B38] Edwards J. Johnson C. Santos-Medellín C. Lurie E. Podishetty N. K. Bhatnagar S. (2015). Structure, variation, and assembly of the root-associated microbiomes of rice. *Proc. Natl. Acad. Sci. U.S.A*, 112 E911–E920. 25605935 10.1073/pnas.1414592112 PMC4345613

[B39] Eyre A. W. Wang M. Oh Y. Dean R. A. (2019). Identification and characterization of the core rice seed microbiome. *Phytobio. J.* 3 148–157. 10.1094/pbiomes-01-19-0009-r

[B40] Ferreira A. Quecine M. C. Lacava P. T. Oda S. Azevedo J. L. Araújo W. L. (2008). Diversity of endophytic bacteria from Eucalyptus species seeds and colonization of seedlings by Pantoea agglomerans. *FEMS Microbiol. Lett.* 287 8–14. 18710397 10.1111/j.1574-6968.2008.01258.x

[B41] Fitzpatrick C. R. Copeland J. Wang P. W. Guttman D. S. Kotanen P. M. Johnson M. T. J. (2018). Assembly and ecological function of the root microbiome across angiosperm plant species. *Proc. Natl. Acad. Sci. U.S.A.* 115 E1157–E1165. 10.1073/pnas.1717617115 29358405 PMC5819437

[B42] Fulthorpe R. Martin A. R. Isaac M. E. (2020). Root endophytes of coffee (*Coffea arabica*): variation across climatic gradients and relationships with functional traits. *Phytobio. J.* 4 27–39. 10.1094/pbiomes-04-19-0021-r

[B43] Gaston K. J. Blackburn T. M. Greenwood J. J. D. Gregory R. D. Quinn R. M. Lawton J. H. (2000). Abundance–occupancy relationships. *J. Appl. Ecol.* 37 39–59. 10.1046/j.1365-2664.2000.00485.x

[B44] Glynou K. Ali T. Buch A. K. Haghi Kia S. Ploch S. Xia X. (2016). The local environment determines the assembly of root endophytic fungi at a continental scale. *Environ. Microbiol.* 18 2418–2434. 26530450 10.1111/1462-2920.13112

[B45] Glynou K. Thines M. Maciá-Vicente J. G. (2018). Host species identity in annual Brassicaceae has a limited effect on the assembly of root-endophytic fungal communities. *Plant Ecol. Divers.* 11 569–580. 10.1080/17550874.2018.1504332

[B46] Grady K. L. Sorensen J. W. Stopnisek N. Guittar J. Shade A. (2019). Assembly and seasonality of core phyllosphere microbiota on perennial biofuel crops. *Nat. Commun.* 10:4135. 10.1038/s41467-019-11974-4 31515535 PMC6742659

[B47] Hallmann J. Berg G. (2006). “Spectrum and population dynamics of bacterial root endophytes,” in *Microbial Root Endophytes*, eds Schulz B. J. E. Boyle C. J. C. Sieber T. N. (Berlin: Springer), 15–31.

[B48] Hamonts K. Trivedi P. Garg A. Janitz C. Grinyer J. Holford P. (2018). Field study reveals core plant microbiota and relative importance of their drivers. *Environ. Microbiol.* 20 124–140. 10.1111/1462-2920.14031 29266641

[B49] Hardoim P. (2019). “The ecology of seed microbiota,” in *Seed Endophytes: Biology and Biotechnology*, eds Verma S. K. White J. J. F. (Cham: Springer International Publishing), 103–125.

[B50] Hardoim P. R. Hardoim C. C. Van Overbeek L. S. Van Elsas J. D. (2012). Dynamics of seed-borne rice endophytes on early plant growth stages. *PloS One* 7:e30438. 10.1371/journal.pone.0030438 22363438 PMC3281832

[B51] Higgins K. L. Arnold A. E. Coley P. D. Kursar T. A. (2014). Communities of fungal endophytes in tropical forest grasses: highly diverse host- and habitat generalists characterized by strong spatial structure. *Fungal Ecol.* 8 1–11. 10.1016/j.funeco.2013.12.005

[B52] Hong S. Bunge J. Leslin C. Jeon S. Epstein S. S. (2009). Polymerase chain reaction primers miss half of rRNA microbial diversity. *ISME J.* 3 1365–1373. 10.1038/ismej.2009.89 19693101

[B53] Horton M. W. Bodenhausen N. Beilsmith K. Meng D. Muegge B. D. Subramanian S. (2014). Genome-wide association study of Arabidopsis thaliana leaf microbial community. *Nat. Commun.* 5:5320. 10.1038/ncomms6320 25382143 PMC4232226

[B54] Ibáñez F. Tonelli M. L. Muñoz V. Figueredo M. S. Fabra A. (2017). “Bacterial endophytes of plants: diversity, invasion mechanisms and effects on the host,” in *Endophytes: Biology and Biotechnology*, Vol. 1 ed. Maheshwari D. K. (Cham: Springer International Publishing), 25–40.

[B55] Inceoğlu Ö Al-Soud W. A. Salles J. F. Semenov A. V. van Elsas J. D. (2011). Comparative analysis of bacterial communities in a potato field as determined by pyrosequencing. *PLoS One* 6:e23321. 10.1371/journal.pone.0023321 21886785 PMC3158761

[B56] Johnston-Monje D. Arévalo A. L. Bolaños A. C. (2021). “Friends in low places: Soil derived microbial inoculants for biostimulation and biocontrol in crop production,” in *Microbiome Stimulants for Crops*, eds White J. F. Kumar A. Droby S. (Amsterdam: Elsevier), 15–31.

[B57] Johnston-Monje D. Loewen S. Lazarovits G. (2017). Mycobiomes of tomato plants with vine decline. *Can. J. Plant Pathol.* 39 184–200. 10.1080/07060661.2017.1325938

[B58] Johnston-Monje D. Lundberg D. S. Lazarovits G. Reis V. M. Raizada M. N. (2016). Bacterial populations in juvenile maize rhizospheres originate from both seed and soil. *Plant Soil* 405 337–355. 10.1007/s11104-016-2826-0

[B59] Johnston-Monje D. Mousa W. K. Lazarovits G. Raizada M. N. (2014). Impact of swapping soils on the endophytic bacterial communities of pre-domesticated, ancient and modern maize. *BMC Plant Biol.* 14:233. 10.1186/s12870-014-0233-3 25227492 PMC4189167

[B60] Johnston-Monje D. Raizada M. N. (2011). Conservation and diversity of seed associated endophytes in Zea across boundaries of evolution, ethnography and ecology. *PLoS One* 6:e20396. 10.1371/journal.pone.0020396 21673982 PMC3108599

[B61] Jumpponen A. Herrera J. Porras-Alfaro A. Rudgers J. (2017). “Biogeography of root-associated fungal endophytes,” in *Biogeography of Mycorrhizal Symbiosis*, ed. Tedersoo L. (Cham: Springer International Publishing), 195–222.

[B62] Kaga H. Mano H. Tanaka F. Watanabe A. Kaneko S. Morisaki H. (2009). Rice seeds as sources of endophytic bacteria. *Microbes Environ.* 24 154–162. 21566368 10.1264/jsme2.me09113

[B63] Kawasaki A. Donn S. Ryan P. R. Mathesius U. Devilla R. Jones A. (2016). Microbiome and exudates of the root and rhizosphere of brachypodium distachyon, a model for wheat. *PLoS One* 11:e0164533. 10.1371/journal.pone.0164533 27727301 PMC5058512

[B64] Klaedtke S. Jacques M.-A. Raggi L. Préveaux A. Bonneau S. Negri V. (2016). Terroir is a key driver of seed-associated microbial assemblages. *Environ. Microbiol.* 18 1792–1804. 10.1111/1462-2920.12977 26171841

[B65] Laur J. Ramakrishnan G. B. Labbé C. Lefebvre F. Spanu P. D. Bélanger R. R. (2018). Effectors involved in fungal–fungal interaction lead to a rare phenomenon of hyperbiotrophy in the tritrophic system biocontrol agent–powdery mildew–plant. *New Phytol.* 217 713–725. 10.1111/nph.14851 29044534 PMC6079639

[B66] Lee S. A. Kim Y. Kim J. M. Chu B. Joa J.-H. Sang M. K. (2019). A preliminary examination of bacterial, archaeal, and fungal communities inhabiting different rhizocompartments of tomato plants under real-world environments. *Sci. Rep.* 9:9300. 10.1038/s41598-019-45660-8 31243310 PMC6594962

[B67] Leff J. W. Lynch R. C. Kane N. C. Fierer N. (2017). Plant domestication and the assembly of bacterial and fungal communities associated with strains of the common sunflower, Helianthus annuus. *New Phytol.* 214 412–423. 10.1111/nph.14323 27879004

[B68] Lemanceau P. Blouin M. Muller D. Moënne-Loccoz Y. (2017). Let the core microbiota be functional. *Trends Plant Sci.* 22 583–595. 28549621 10.1016/j.tplants.2017.04.008

[B69] Li H. Parmar S. Sharma V. K. White J. F. (2019). “Seed endophytes and their potential applications,” in *Seed Endophytes: Biology and Biotechnology*, eds Verma S. K. White J. J. F. (Cham: Springer International Publishing), 35–54.

[B70] Links M. G. Demeke T. Gräfenhan T. Hill J. E. Hemmingsen S. M. Dumonceaux T. J. (2014). Simultaneous profiling of seed-associated bacteria and fungi reveals antagonistic interactions between microorganisms within a shared epiphytic microbiome on Triticum and Brassica seeds. *New Phytol.* 202 542–553. 10.1111/nph.12693 24444052 PMC4235306

[B71] Liu F. Hewezi T. Lebeis S. L. Pantalone V. Grewal P. S. Staton M. E. (2019). Soil indigenous microbiome and plant genotypes cooperatively modify soybean rhizosphere microbiome assembly. *BMC Microbiol.* 19:201. 10.1186/s12866-019-1572-x 31477026 PMC6720100

[B72] López-López A. Rogel M. A. Ormeno-Orrillo E. Martínez-Romero J. Martínez-Romero E. (2010). Phaseolus vulgaris seed-borne endophytic community with novel bacterial species such as Rhizobium endophyticum sp. nov. *Syst. Appl. Microbiol.* 33 322–327. 20822874 10.1016/j.syapm.2010.07.005

[B73] Lundberg D. S. Lebeis S. L. Paredes S. H. Yourstone S. Gehring J. Malfatti S. (2012). Defining the core Arabidopsis thaliana root microbiome. *Nature* 488 86–90. 22859206 10.1038/nature11237 PMC4074413

[B74] Lundberg D. S. Yourstone S. Mieczkowski P. Jones C. D. Dangl J. L. (2013). Practical innovations for high-throughput amplicon sequencing. *Nat. Methods* 10:999. 10.1038/nmeth.2634 23995388

[B75] Maccarone L. (2013). Relationships between the pathogen *Olpidium virulentus* and viruses associated with lettuce big-vein disease. *Plant Dis.* 97 700–707. 30722639 10.1094/PDIS-10-12-0979-FE

[B76] Mano H. Tanaka F. Watanabe A. Kaga H. Okunishi S. Morisaki H. (2006). Culturable surface and endophytic bacterial flora of the maturing seeds of rice plants (*Oryza sativa*) cultivated in a paddy field. *Microbes Environ.* 21 86–100.

[B77] McKnight D. T. Huerlimann R. Bower D. S. Schwarzkopf L. Alford R. A. Zenger K. R. (2019). Methods for normalizing microbiome data: an ecological perspective. *Methods Ecol. Evol.* 10 389–400. 10.1111/2041-210X.13115

[B78] Mechan-Llontop M. E. Tian L. Sharma P. Heflin L. Bernal-Galeano V. Haak D. C. (2021). Experimental evidence pointing to rain as a reservoir of tomato phyllosphere microbiota. *Biorxiv*, [Preprint] 10.1101/2021.04.08.438997

[B79] Michielse C. B. Rep M. (2009). Pathogen profile update: *Fusarium oxysporum*. *Mol. Plant Pathol.* 10 311–324. 10.1111/j.1364-3703.2009.00538.x 19400835 PMC6640313

[B80] Mirmajlessi S. M. Bahram M. Mänd M. Najdabbasi N. Mansouripour S. Loit E. (2018). Survey of soil fungal communities in strawberry fields by illumina amplicon sequencing. *Eur. Soil Sci.* 51 682–691. 10.1134/S106422931806011X

[B81] Moreira Z. P. M. Helgason B. L. Germida J. J. (2021). Crop, genotype, and field environmental conditions shape bacterial and fungal seed epiphytic microbiomes. *Can. J. Microbiol.* 67 161–173. 10.1139/cjm-2020-0306 32931717

[B82] Müller C. A. Obermeier M. M. Berg G. (2016). Bioprospecting plant-associated microbiomes. *J. Biotechnol.* 235 171–180. 10.1016/j.jbiotec.2016.03.033 27015976

[B83] Mundt J. O. Hinkle N. F. (1976). Bacteria within ovules and seeds. *Appl. Environ. Microbiol.* 32 694–698. 984839 10.1128/aem.32.5.694-698.1976 PMC170385

[B84] Nasanit R. Tangwong-o-thai A. Tantirungkij M. Limtong S. (2015b). The assessment of epiphytic yeast diversity in sugarcane phyllosphere in Thailand by culture-independent method. *Fungal Biol.* 119 1145–1157. 10.1016/j.funbio.2015.08.021 26615738

[B85] Nasanit R. Krataithong K. Tantirungkij M. Limtong S. (2015a). Assessment of epiphytic yeast diversity in rice (*Oryza sativa*) phyllosphere in Thailand by a culture-independent approach. *Ant. Van Leeuwen.* 107 1475–1490. 10.1007/s10482-015-0442-2 25842038

[B86] Nebert L. D. (2018). *On Germs and Germination: Uncovering the Hidden Ecology of Seedborne Bacteria and Fungi in Open-Pollinated Maize.* Oregon, OR: University of Oregon.

[B87] Nelson E. B. (2004). Microbial dynamics and interactions in the spermosphere. *Annu. Rev. Phytopathol.* 42 271–309. 15283668 10.1146/annurev.phyto.42.121603.131041

[B88] Nelson E. B. (2018). The seed microbiome: origins, interactions, and impacts. *Plant Soil* 422 7–34.

[B89] Niu B. Paulson J. N. Zheng X. Kolter R. (2017). Simplified and representative bacterial community of maize roots. *Proc. Natl. Acad. Sci. U.S.A.* 114 E2450–E2459. 10.1073/pnas.1616148114 28275097 PMC5373366

[B90] O’Callaghan M. (2016). Microbial inoculation of seed for improved crop performance: issues and opportunities. *Appl. Microbiol. Biotechnol.* 100 5729–5746. 10.1007/s00253-016-7590-9 27188775 PMC4909795

[B91] O’Donnell K. Ward T. J. Robert V. A. R. G. Crous P. W. Geiser D. M. Kang S. (2015). DNA sequence-based identification of *Fusarium*: current status and future directions. *Phytoparasitica* 43 583–595. 10.1007/s12600-015-0484-z

[B92] Ortega R. A. Mahnert A. Berg C. Müller H. Berg G. (2016). The plant is crucial: specific composition and function of the phyllosphere microbiome of indoor ornamentals. *FEMS Microbiol. Ecol.* 92:fiw173. 10.1093/femsec/fiw173 27624084

[B93] Ottesen A. R. Gorham S. Reed E. Newell M. J. Ramachandran P. Canida T. (2016). Using a control to better understand phyllosphere microbiota. *PLoS One* 11:e0163482. 10.1371/journal.pone.0163482 27669159 PMC5036865

[B94] Peay K. G. Kennedy P. G. Talbot J. M. (2016). Dimensions of biodiversity in the Earth mycobiome. *Nat. Rev. Microbiol.* 14 434–447. 10.1038/nrmicro.2016.59 27296482

[B95] Peiffer J. A. Spor A. Koren O. Jin Z. Tringe S. G. Dangl J. L. (2013). Diversity and heritability of the maize rhizosphere microbiome under field conditions. *Proc. Natl. Acad. Sci. U.S.A.* 110 6548–6553. 10.1073/pnas.1302837110 23576752 PMC3631645

[B96] Pérez-Jaramillo J. E. de Hollander M. Ramírez C. A. Mendes R. Raaijmakers J. M. Carrión V. J. (2019). Deciphering rhizosphere microbiome assembly of wild and modern common bean (*Phaseolus vulgaris*) in native and agricultural soils from Colombia. *Microbiome* 7:114. 10.1186/s40168-019-0727-1 PMC6694607 31412927

[B97] Pfeiffer S. Mitter B. Oswald A. Schloter-Hai B. Schloter M. Declerck S. (2017). Rhizosphere microbiomes of potato cultivated in the high andes show stable and dynamic core microbiomes with different responses to plant development. *FEMS Microbiol. Ecol.* 93:fiw242. 10.1093/femsec/fiw242 27940644

[B98] Pollock J. Glendinning L. Wisedchanwet T. Watson M. (2018). The madness of microbiome: attempting to find consensus “best practice” for 16s microbiome studies. *Appl. Environ. Microbiol.* 84 e2627–e2617. 10.1128/AEM.02627-17 29427429 PMC5861821

[B99] Porras-Alfaro A. Bayman P. (2011). Hidden fungi, emergent properties: endophytes and microbiomes. *Annu. Rev. Phytopathol.* 49 291–315. 10.1146/annurev-phyto-080508-081831 19400639

[B100] Puente M. E. Li C. Y. Bashan Y. (2009). Endophytic bacteria in cacti seeds can improve the development of cactus seedlings. *Environ. Exp. Bot.* 66 402–408.

[B101] Rahman M. M. Flory E. Koyro H.-W. Abideen Z. Schikora A. Suarez C. (2018). Consistent associations with beneficial bacteria in the seed endosphere of barley (*Hordeum vulgare L*.). *Syst. Appl. Microbiol.* 41 386–398. 29567394 10.1016/j.syapm.2018.02.003

[B102] Rastogi G. Sbodio A. Tech J. J. Suslow T. V. Coaker G. L. Leveau J. H. J. (2012). Leaf microbiota in an agroecosystem: spatiotemporal variation in bacterial community composition on field-grown lettuce. *ISME J.* 6 1812–1822. 10.1038/ismej.2012.32 22534606 PMC3446804

[B103] Ricks K. D. Koide R. T. (2019). Biotic filtering of endophytic fungal communities in *Bromus tectorum*. *Oecologia* 189 993–1003. 10.1007/s00442-019-04388-y 30900053

[B104] Rochefort A. Simonin M. Marais C. Guillerm-Erckelboudt A.-Y. Barret M. Sarniguet A. (2021). Transmission of Seed and Soil Microbiota to Seedling. *mSystems* 6:e0044621. 10.1128/mSystems.00446-21 34100639 PMC8269233

[B105] Rosenberg E. Zilber-Rosenberg I. (2016). Microbes drive evolution of animals and plants: the hologenome concept. *MBio* 7:e01395. 10.1128/mBio.01395-15 PMC4817260 27034283

[B106] Sadeghi F. Samsampour D. Seyahooei M. A. Bagheri A. Soltani J. (2019). Diversity and spatiotemporal distribution of fungal endophytes associated with citrus reticulata cv. siyahoo. *Curr. Microbiol.* 76 279–289. 10.1007/s00284-019-01632-9 30689005

[B107] Sapkota R. Knorr K. Jørgensen L. N. O’Hanlon K. A. Nicolaisen M. (2015). Host genotype is an important determinant of the cereal phyllosphere mycobiome. *New Phytol.* 207 1134–1144. 10.1111/nph.13418 25898906

[B108] Schlaeppi K. Dombrowski N. Oter R. G. Ver Loren van Themaat E. Schulze-Lefert P. (2014). Quantitative divergence of the bacterial root microbiota in *Arabidopsis thaliana* relatives. *Proc. Natl. Acad. Sci. U.S.A.* 111 585–592. 10.1073/pnas.1321597111 24379374 PMC3896156

[B109] Schlatter D. C. Yin C. Hulbert S. Paulitz T. C. (2020). Core rhizosphere microbiomes of dryland wheat are influenced by location and land use history. *Appl. Environ. Microbiol.* 86 e2135–e2119. 10.1128/aem.02135-19 31862727 PMC7028972

[B110] Schlemper T. R. Leite M. F. A. Lucheta A. R. Shimels M. Bouwmeester H. J. van Veen J. A. (2017). Rhizobacterial community structure differences among sorghum cultivars in different growth stages and soils. *FEMS Microbiol. Ecol.* 93:8. 10.1093/femsec/fix096 28830071

[B111] Schneider C. A. Rasband W. S. Eliceiri K. W. (2012). NIH Image to ImageJ: 25 years of image analysis. *Nat. Methods* 9 671–675. 22930834 10.1038/nmeth.2089 PMC5554542

[B112] Schreiter S. Ding G.-C. Heuer H. Neumann G. Sandmann M. Grosch R. (2014). Effect of the soil type on the microbiome in the rhizosphere of field-grown lettuce. *Front. Microbiol.* 5:144. 10.3389/fmicb.2014.00144 24782839 PMC3986527

[B113] Sessitsch A. Coenye T. Sturz A. V. Vandamme P. Barka E. A. Salles J. F. (2005). Burkholderia phytofirmans sp. nov., a novel plant-associated bacterium with plant-beneficial properties. *Int. J. Syst. Evol. Microbiol.* 55 1187–1192. 10.1099/ijs.0.63149-0 15879253

[B114] Shade A. Handelsman J. (2012). Beyond the Venn diagram: the hunt for a core microbiome. *Environ. Microbiol.* 14 4–12. 10.1111/j.1462-2920.2011.02585.x 22004523

[B115] Shade A. Jones S. E. Caporaso J. G. Handelsman J. Knight R. Fierer N. (2014). Conditionally rare taxa disproportionately contribute to temporal changes in microbial diversity. *Mbio* 5 e1371–e1314. 10.1128/mBio.01371-14 25028427 PMC4161262

[B116] Shade A. Stopnisek N. (2019). Abundance-occupancy distributions to prioritize plant core microbiome membership. *Curr. Opin. Microbiol.* 49 50–58. 10.1016/j.mib.2019.09.008 31715441

[B117] Sharaf H. Rodrigues R. R. Moon J. Zhang B. Mills K. Williams M. A. (2019). Unprecedented bacterial community richness in soybean nodules vary with cultivar and water status. *Microbiome* 7:63. 10.1186/s40168-019-0676-8 30992078 PMC6469096

[B118] Sheibani-Tezerji R. Naveed M. Jehl M.-A. Sessitsch A. Rattei T. Mitter B. (2015). The genomes of closely related Pantoea ananatis maize seed endophytes having different effects on the host plant differ in secretion system genes and mobile genetic elements. *Front. Microbiol.* 6:440. 10.3389/fmicb.2015.00440 26029184 PMC4428218

[B119] Simonin M. Dasilva C. Terzi V. Ngonkeu E. L. M. Diouf D. Kane A. (2020). Influence of plant genotype and soil on the wheat rhizosphere microbiome: evidences for a core microbiome across eight African and European soils. *FEMS Microbiol. Ecol.* 96:fiaa067. 10.1093/femsec/fiaa067 32275297

[B120] Singhal U. Prasad R. Varma A. (2017). “Piriformospora indica (*Serendipita indica*): the novel symbiont,” in *Mycorrhiza-Function, Diversity, State of the Art*, eds Varma A. Prasad R. Tuteja N. (Cham: Springer International Publishing), 349–364.

[B121] Smalla K. (2003). “Culture-independent microbiology,” in *Microbial Diversity and Bioprospecting*, ed. Bull A. T. (Hoboken, NJ: John Wiley & Sons Inc), 88–99.

[B122] Smith D. P. Peay K. G. Ackermann G. Apprill A. Bauer M. Berg-Lyons D. (2020). *Earth Microbiome Project ITS Illumina Amplicon Protocol.* Available online at: https://www.protocols.io/view/emp-its-illumina-amplicon-protocol-pa7dihn (accessed April 21, 2020).

[B123] Stanghellini M. Mathews D. Misaghi I. (2010). Pathogenicity and management of Olpidium bornovanus, a root pathogen of melons. *Plant Dis.* 94 163–166. 30754255 10.1094/PDIS-94-2-0163

[B124] Terrazas R. A. Balbirnie-Cumming K. Morris J. Hedley P. Russell J. Paterson E. (2020). A footptrint of plant eco-geographic adaptation on the composition of the barley rhizosphere bacterial microbiota. *Sci. Rep.* 10:12916. 10.1038/s41598-020-69672-x PMC7395104 32737353

[B125] Thapa S. Prasanna R. (2018). Prospecting the characteristics and significance of the phyllosphere microbiome. *Anna. Microbiol.* 68 229–245. 10.1007/s13213-018-1331-5

[B126] Toju H. Peay K. G. Yamamichi M. Narisawa K. Hiruma K. Naito K. (2018). Core microbiomes for sustainable agroecosystems. *Nat. Plants* 4 247–257. 29725101 10.1038/s41477-018-0139-4

[B127] Truyens S. Beckers B. Thijs S. Weyens N. Cuypers A. Vangronsveld J. (2016). The effects of the growth substrate on cultivable and total endophytic assemblages of *Arabidopsis thaliana*. *Plant Soil* 405 325–336. 10.1007/s11104-015-2761-5

[B128] Truyens S. Weyens N. Cuypers A. Vangronsveld J. (2013). Changes in the population of seed bacteria of transgenerationally Cd-exposed A rabidopsis thaliana. *Plant Biol.* 15 971–981. 23252960 10.1111/j.1438-8677.2012.00711.x

[B129] Truyens S. Weyens N. Cuypers A. Vangronsveld J. (2015). Bacterial seed endophytes: genera, vertical transmission and interaction with plants. *Environ. Microbiol. Rep.* 7 40–50.

[B130] Turner T. R. Ramakrishnan K. Walshaw J. Heavens D. Alston M. Swarbreck D. (2013). Comparative metatranscriptomics reveals kingdom level changes in the rhizosphere microbiome of plants. *ISME J.* 7 2248–2258. 10.1038/ismej.2013.119 23864127 PMC3834852

[B131] Vandenkoornhuyse P. Quaiser A. Duhamel M. Le Van A. Dufresne A. (2015). The importance of the microbiome of the plant holobiont. *New Phytol.* 206 1196–1206. 10.1111/nph.13312 25655016

[B132] Verma S. White J. (2018). Indigenous endophytic seed bacteria promote seedling development and defend against fungal disease in browntop millet (*Urochloa ramosa L*.). *J. Appl. Microbiol.* 124 764–778. 29253319 10.1111/jam.13673

[B133] von-Maltzahn G. Flavell R. B. Toledo G. V. Djonovic S. Márquez L. M. Johnston D. M. (2015). *Seed-Origin Endophyte Populations, Compositions, and Methods of Use (US9113636B2).* Cambridge, MA: Digital Science & Research Solutions Inc.

[B134] von-Maltzahn G. Flavell R. B. Toledo G. V. Djonovic S. Márquez L. M. Johnston D. M. (2017). *Methods of Use of Seed-Origin Endophyte Populations (US9532572B2).* Boston, MA: Indigo Ag Inc.

[B135] Wagner M. R. Lundberg D. S. del Rio T. G. Tringe S. G. Dangl J. L. Mitchell-Olds T. (2016). Host genotype and age shape the leaf and root microbiomes of a wild perennial plant. *Nat. Commun.* 7:12151. 10.1038/ncomms12151 27402057 PMC4945892

[B136] Wallace J. G. Kremling K. A. Kovar L. L. Buckler E. S. (2018). Quantitative genetics of the maize leaf microbiome. *Phytobio. J.* 2 208–224. 10.1094/pbiomes-02-18-0008-r

[B137] Walsh C. M. Becker-Uncapher I. Carlson M. Fierer N. (2021). Variable influences of soil and seed-associated bacterial communities on the assembly of seedling microbiomes. *ISME J.* 15 2748–2762. 33782567 10.1038/s41396-021-00967-1 PMC8397733

[B138] Walters W. A. Jin Z. Youngblut N. Wallace J. G. Sutter J. Zhang W. (2018). Large-scale replicated field study of maize rhizosphere identifies heritable microbes. *Proc. Natl. Acad. Sci. U.S.A.* 115 7368–7373. 10.1073/pnas.1800918115 29941552 PMC6048482

[B139] Wang B. Sugiyama S. (2020). Phylogenetic signal of host plants in the bacterial and fungal root microbiomes of cultivated angiosperms. *Plant J.* 104 522–531. 32744366 10.1111/tpj.14943

[B140] Wang P. Kong X. Chen H. Xiao Y. Liu H. Li X. (2021). Exploration of intrinsic microbial community modulators in the rice endosphere indicates a key role of distinct bacterial taxa across different cultivars. *Front. Microbiol.* 12:629852. 10.3389/fmicb.2021.629852 33664718 PMC7920960

[B141] Wassermann B. Adam E. Cernava T. Berg G. (2019a). “Understanding the indigenous seed microbiota to design bacterial seed treatments,” in *Seed Endophytes: Biology and Biotechnology*, eds Verma S. K. White J. J. F. (Cham: Springer International Publishing), 83–99.

[B142] Wassermann B. Cernava T. Müller H. Berg C. Berg G. (2019b). Seeds of native alpine plants host unique microbial communities embedded in cross-kingdom networks. *Microbiome* 7:108. 10.1186/s40168-019-0723-5 31340847 PMC6651914

[B143] Weller D. M. Raaijmakers J. M. Gardener B. B. M. Thomashow L. S. (2002). Microbial populations responsible for specific soil suppressiveness to plant pathogens. *Annu. Rev. Phytopathol.* 40 309–348. 10.1146/annurev.phyto.40.030402.110010 12147763

[B144] Whipps J. Hand P. Pink D. Bending G. D. (2008). Phyllosphere microbiology with special reference to diversity and plant genotype. *J. Appl. Microbiol.* 105 1744–1755. 19120625 10.1111/j.1365-2672.2008.03906.x

[B145] White J. F. Chen Q. Torres M. S. Mattera R. Irizarry I. Tadych M. (2015). Collaboration between grass seedlings and rhizobacteria to scavenge organic nitrogen in soils. *AOB Plants* 7 lu093–lu093. 10.1093/aobpla/plu093 25564515 PMC4313791

[B146] White J. F. Kingsley K. L. Verma S. K. Kowalski K. P. (2018). Rhizophagy cycle: an oxidative process in plants for nutrient extraction from symbiotic microbes. *Microorganisms* 6:95. 10.3390/microorganisms6030095 PMC6164190 30227634

[B147] Williams T. R. Marco M. L. (2014). Phyllosphere microbiota composition and microbial community transplantation on lettuce plants grown indoors. *mBio.* 5 e1564–e1514. 10.1128/mBio.01564-14 25118240 PMC4145687

[B148] Xu L. Nicolaisen M. Larsen J. Zeng R. Gao S. Dai F. (2020). Pathogen infection and host-resistance interactively affect root-associated fungal communities in watermelon. *Front. Microbiol.* 11:605622. 10.3389/fmicb.2020.605622 33424807 PMC7793699

[B149] Yang L. Danzberger J. Schöler A. Schröder P. Schloter M. Radl V. (2017). Dominant groups of potentially active bacteria shared by barley seeds become less abundant in root associated microbiome. *Front. Plant Sci.* 8:1005. 10.3389/fpls.2017.01005 28663753 PMC5471333

[B150] Yeoh Y. K. Dennis P. G. Paungfoo-Lonhienne C. Weber L. Brackin R. Ragan M. A. (2017). Evolutionary conservation of a core root microbiome across plant phyla along a tropical soil chronosequence. *Nat. Commun.* 8:215. 10.1038/s41467-017-00262-8 28790312 PMC5548757

[B151] Yeoh Y. K. Paungfoo-Lonhienne C. Dennis P. G. Robinson N. Ragan M. A. Schmidt S. (2016). The core root microbiome of sugarcanes cultivated under varying nitrogen fertilizer application. *Environ. Microbiol.* 18 1338–1351. 26032777 10.1111/1462-2920.12925

[B152] Zarraonaindia I. Owens S. M. Weisenhorn P. West K. Hampton-Marcell J. Lax S. (2015). The soil microbiome influences grapevine-associated microbiota. *MBio* 6:e02527. 10.1128/mBio.02527-14 PMC4453523 25805735

